# Subsequent biotic crises delayed marine recovery following the late Permian mass extinction event in northern Italy

**DOI:** 10.1371/journal.pone.0172321

**Published:** 2017-03-15

**Authors:** William J. Foster, Silvia Danise, Gregory D. Price, Richard J. Twitchett

**Affiliations:** 1 Jackson School of Geosciences, University of Texas, Austin, Texas, United States of America; 2 Earth Sciences, Plymouth University, Plymouth, United Kingdom; 3 Dept. of Geology, University of Georgia, Athens, Georgia, United States of America; 4 Dept. of Earth Sciences, Natural History Museum, London, United Kingdom; Naturhistoriska riksmuseet, SWEDEN

## Abstract

The late Permian mass extinction event was the largest biotic crisis of the Phanerozoic and has the longest recovery interval of any extinction event. It has been hypothesised that subsequent carbon isotope perturbations during the Early Triassic are associated with biotic crises that impeded benthic recovery. We test this hypothesis by undertaking the highest-resolution study yet made of the rock and fossil records of the entire Werfen Formation, Italy. Here, we show that elevated extinction rates were recorded not only in the Dienerian, as previously recognised, but also around the Smithian/Spathian boundary. Functional richness increases across the Smithian/Spathian boundary associated with elevated origination rates in the lower Spathian. The taxonomic and functional composition of benthic faunas only recorded two significant changes: (1) reduced heterogeneity in the Dienerian, and (2) and a faunal turnover across the Smithian/Spathian boundary. The elevated extinctions and compositional shifts in the Dienerian and across the Smithian/Spathian boundary are associated with a negative and positive isotope excursion, respectively, which supports the hypothesis that subsequent biotic crises are associated with carbon isotope shifts. The Spathian fauna represents a more advanced ecological state, not recognised in the previous members of the Werfen Formation, with increased habitat differentiation, a shift in the dominant modes of life, appearance of stenohaline taxa and the occupation of the erect and infaunal tiers. In addition to subsequent biotic crises delaying the recovery, therefore, persistent environmental stress limited the ecological complexity of benthic recovery prior to the Spathian.

## Introduction

The late Permian mass extinction event is the most catastrophic crisis to have affected life during the Phanerozoic, with a loss of an estimated 81% of marine species[1], and is associated with climate-induced environmental changes triggered by Siberian Traps volcanism[2–3]. Previous studies have shown that modest benthic recovery is recorded within the Griesbachian, i.e. first Triassic substage (e.g.[4–5]). Yet, the final stage of recovery is typically not recorded until the Middle Triassic (e.g.[6–7]). Geochemical data shows that the Early Triassic is characterised by multiple carbon and oxygen isotope excursions with the late Griesbachian and Smithian/Spathian boundary (SSB) recording thermal maxima[8], which may have resulted in further environmental deterioration that delayed recovery from the extinction event[9].

Benthic biotic crises have been recorded at the Griesbachian/Dienerian boundary in Oman[5], and during the Dienerian in western US [10] and the Werfen Formation, Italy[11], but not at all locations [12]. Some cosmopolitan benthic taxa become globally extinct at the SSB, e.g. bellerophontids[13], and some functional groups declined in relative abundance globally[14], but there is no evidence that marine ecosystem recovery was significantly impeded. A ‘brief reversal’ in regional recovery was recorded in the Smithian Campil Member of the Werfen Formation, northern Italy, but attributed to local facies change[11,15–16]. Similarly, turnovers in the taxonomic and functional composition of benthic assemblages across the SSB identified in Hungary [12] and western US [17] are due, at least in part, to differences in sampled facies. Evidence that the subsequent hyperthermals delayed the recovery of the benthos is, therefore, equivocal.

The main aim of this study is to test whether carbon isotope perturbations are associated with biotic crisis that delayed the recovery of benthic marine invertebrates. In particular, we aim to quantitatively assess how (i) alpha diversity and (ii) the taxonomic and functional composition of benthic communities change through the studied interval. Here, we present the highest-resolution and most continuous quantitative dataset yet assembled from the entire Lower Triassic Werfen Formation, Italy, which allows a better control on the impact of facies-induced bias on the interpretation of ecological changes. In addition, changes in species richness and functional richness were analysed separately as environmental degradation can reduce the functional diversity of animal communities beyond changes in species richness alone[18]. The continuous, easily accessible, fossiliferous record, within a succession of repeated facies that help minimise facies-induced bias, and a well-established bio-, litho-, and chemostratigraphic framework, make the Werfen Formation ideal for testing this hypothesis.

### Geological and stratigraphical setting

**Fig 1:**
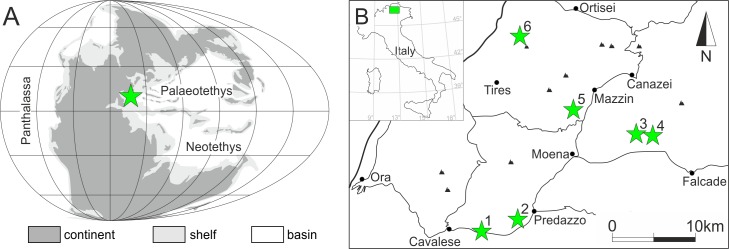
Location maps of the study sites. **A)** Palaeogeographic map of the Early Triassic after [23] indicating approximate position of the Dolomites, Italy. **B)** Location of the investigated sections: 1—Tesero; 2—Val Averta; 3—Costabella; 4—l’Uomo; 5—Rio di Pantl; 6—Siusi.

**Fig 2:**
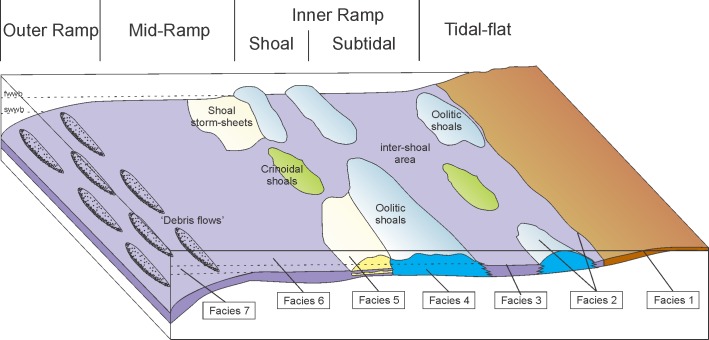
**Schematic facies interpretation for the Werfen Formation, Italy;** Facies: 1 –supratidal, 2 –peritidal, 3 –shallow subtidal, inner ramp, 4 –shoal, 5 –mid-ramp with oolitic storm sheets, 6 –mid-ramp with storm sheets, 7 –outer ramp with ‘debris flows’. For a definition of facies see S1 Table.

The Lower Triassic succession of the Dolomites, Italy, is represented by the Werfen Formation which is approximately 250m thick in the Adige Valley and up to 600-700m thick in the eastern Dolomites[19]. In places, erosion during the Middle Triassic has removed the upper part of the Werfen Formation and is unconformably overlain by the late Anisian (Pelsonian-Illyrian) Richthofen Conglomerate, whereas elsewhere it is complete and conformably overlaid by the Lower Serla Dolomite Formation[20]. During the Early Triassic, the depositional area of the Werfen Formation comprised a segment of the western Palaeotethyan shelf at a low northerly latitude (Fig 1[21]) and deposition took place in a mixed carbonate-siliciclastic homoclinal ramp setting (Fig 2). Detailed descriptions of the facies and shelf evolution of the Lower Triassic succession are given by[22], who recorded four main transgressive-regressive depositional cycles from outer ramp to supratidal settings.

**Fig 3:**
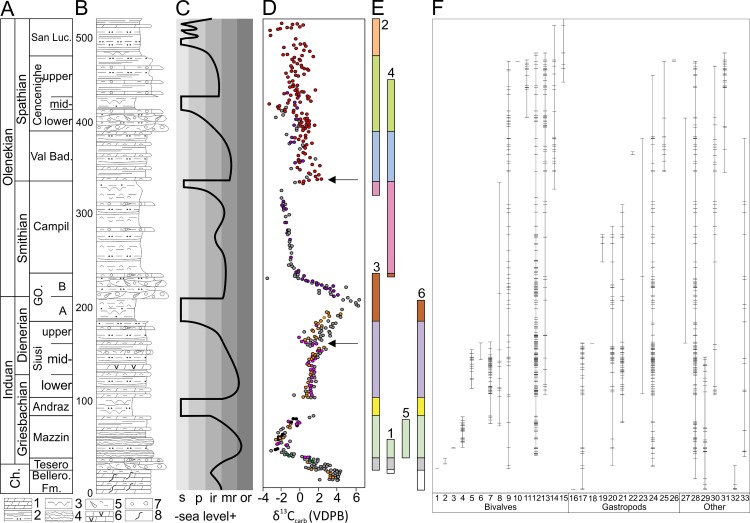
Stratigraphic framework and composite stratigraphic range data for invertebrate taxa for the Werfen Formation, Dolomites. **A)** Lower Triassic substages and members of the Werfen Formation following[24]. Vertical subdivision is proportional to thickness after[24]. Ch.—Changhsingian, Bellero. Fm—Bellerophon Formation, GO.—Gastropod Oolite, Val Bad.—Val Badia, San Luc.—San Lucano. **B)** Lithological column, 1 –limestone facies; 2—clastic facies; 3—tepee structures; 4 –debris-flow facies; 5—bioclasts; 6 –vuggy dolomite; 7—ooids; 8 –bioturbation. **C)** Sea level curve. s—supratidal, p—intertidal, ir—inner ramp, mr—mid-ramp, or–outer ramp. **D)** Bulk carbonate carbon isotopes. Colored dots: this study (green—Tesero, black—Rio Di Pantl, orange—Siusi, pink—l’Uomo, purple—Costabella, red—Val Averta), grey dots after [25–26]. The Dienerian and SSB events discussed in the text are highlighted by arrows. **E)** Investigated sections. 1—Tesero; 2—Val Averta; 3 –l’Uomo; 4—Costabella; 5—Rio di Pantl; 6 –Siusi. **F)** Ranges of benthic invertebrates (this study). Horizontal lines represent occurrences. Species numbers are listed in S1 Text.

Six stratigraphic sections of the Werfen Formation in the Dolomites, Italy, were studied: Tesero, Val Averta, l’Uomo, Costabella, Rio di Pantl and Siusi (Fig 1B). No single section completely exposes the entire formation, but when combined these sections provide a complete succession from a similar depositional setting, with most of the members investigated at multiple sections (Fig 3). Detailed descriptions of the facies and ramp evolution of the Werfen Formation are given by[19,22]. This study recognised seventeen facies, representing tidal-flat, shallow subtidal, inner ramp, shoal, mid-ramp, and outer ramp depositional environments (Fig 2 and S1 Table).

The Werfen Formation is represented by nine members: Tesero, Mazzin, Andraz, Siusi, Gastropod Oolite, Campil, Val Badia, Cencenighe and San Lucano. Details of their biostratigraphic framework are given by [19–20,24,27–28]. Eight bivalve and six conodont biozones have been determined for the Werfen Formation and four ammonoid biozones are recognised in the Spathian members [24]. In sections across the globe that record continuous deposition across the Permian-Triassic transition, it is evident that the late Permian mass extinction event and the Permian/Triassic boundary do not occur at the same horizon (e.g. [29]). The mass extinction event and the Permian/Triassic boundary in the Werfen Formation have also been observed to occur at different horizons [30]. The conodont *Hindeodus parvus*, which defines the base of the Triassic at the condensed GSSP section in Meishan [29], occurs in the basal Tesero Member but is very rare [27,31], and is first recorded at different stratigraphic heights in different sections. The Tesero Member is a transgressive unit and, coupled with the rarity of *H*. *parvus* and the carbon isotope records, this has resulted in a view that the base of the Werfen Formation is diachronous and the height of the Permian/Triassic boundary above the base of the formation varies across the region (e.g. [32]). The final disappearance of common late Permian taxa during the basal Werfen Formation transgression has been used to identify the extinction event locally (e.g. [19,30,33–34]). The Early Triassic is divided two stages, the Induan and Olenekian, and have both been further divided into two substages. The Induan is divided into the Griesbachian and Dienerian, and the Olenekian is divided into the Smithian and Spathian ([35] Fig 3). Here we investigate the biotic changes using the substage chronostratigraphic scale as the isotopic excursions hypothesised to represent biotic crises occur at the Early Triassic substage boundaries [9]. The G/D boundary occurs at the base of the *Claraia aurita* Biozone between the lower- and mid-Siusi Member [19,24]. Based on an extensive review of the then-available chemostratigraphic and biostratigraphic data, [24] defined the Induan/Olenekian boundary by an isotope peak between units A and B of the Gastropod Oolite Member. The Smithian/Spathian boundary is defined at the base of the *Tirolites cassianus* Zone, which occurs in the lower Val Badia Member [20,28] and is associated with a carbon isotope excursion [25] (Fig 3).

## Methods

Sections were logged in September 2012 and June-July 2013 (S1–S6 Figs), using the formation and unit/member definitions of [19]. Lithologies, sedimentary structures and trace fossils were described for each measured bed. In total, 328 fossiliferous beds were sampled for invertebrate macrofossils. Cemented beds that could not be split easily in the field were analysed in the laboratory following the polished slab technique of [12]. All identifiable fossils in the polished slabs were identified to the most precise taxonomic level to which they could be confidently assigned (see Supplementary Material). Taxonomic resolution varied between fossil groups, ranging from species- to phylum-level (S2 Text). All bioclasts within a 5×5cm quadrat over the polished surface of each sample were identified to measure taxonomic richness and tallied to obtain abundance data. Fissile beds were sampled in the field by splitting 2kg of bulk rock parallel to bedding to reveal the fossils, which were then identified and counted. On exposed, fossiliferous bedding planes all fossils within a randomly placed 20 x 20cm quadrat were identified and counted. The fossil material is stored in the South Tyrol Museum of Natural Sciences, Italy (Naturmuseum Bozen; PZO5795-PZO5979).

Carbon and oxygen isotopes were measured from powders drilled from rock chips collected from fresh rock surfaces from the Val Averta section (analysed at Plymouth University), and from fresh rock surfaces of the polished slabs used in the palaeoecological analysis (analysed at University College London) covering the entire range of carbonate lithologies. Isotopic compositions were measured on CO_2_ gas extracted via H_3_PO_4_ and results were calibrated against NBS-19. The δ^18^O and δ^13^C compositions are reported in per mil (‰) notation with respect to the V-PDB international standard. The reproducibility of replicated standards was typically better than 0.1‰ (one standard deviation) for δ^13^C and δ^18^O.

Palaeoecological analyses were limited to benthic marine invertebrates and used the minimum number of individuals (MNI) method (e.g. [12]), and samples with MNI <20 were removed. As multiple methods were used to collect the data, analyses were carried out using the taxonomic resolution of the polished slab technique, which enabled the different samples to be analysed together. Reducing the taxonomic precision of the bedding plane and mechanical disaggregation data does not significantly reduce species richness (*p =* 0.44) or Simpson diversity (*p =* 0.41). Taxonomic identifications follow previous palaeontological studies of the Werfen Formation of the Dolomites (Table 1), except that the bivalve genus *Unionites* is reassigned to *Austrotindaria* following [36] and for the multivariate analyses *Claraia wangi-griesbachi* and *C*. *aurita* were combined as *Claraia aurita* group following [11]. Functional diversity was measured by assigning each taxon to a bin in the ecospace model of [37] based on its tiering, motility and feeding [14]. Unidentified taxa were assigned to a bin in the ecospace model based on comparisons of their morphology with other known taxa.

**Table 1:** List of all recorded taxa and their mode of life. Modes of life after[14]. T = Tiering: 2 = erect, 3 = epifaunal, 4 = semi-infaunal, 5 = shallow infaunal. M = Motility: 2 = slow, 4 = facultatively motile, attached, 3 = facultatively motile, unattached, 5 = stationary, unattached, 6 = stationary, attached. F = Feeding: 1 = suspension feeder, 2 = surface deposit feeder, 3 = miner, 4 = grazer, 5 = predator (see[37] for definitions of functional modes).

Species	Group	Mode of Life			Identification after
		T	M	F	
Brachiopod sp.	Brachiopod	3	6	1	-
*Lingularia* spp.	Brachiopod	5	4	1	[40]
*Lingularia borealis*	Brachiopod	5	4	1	[40]
*Lingularia yini*	Brachiopod	5	4	1	[40]
Bivalve sp. A	Bivalve	3	6	1	-
Bivalve sp. B	Bivalve	3	6	1	-
Bivalve sp. C	Bivalve	3	6	1	-
cf. *Unionites donacinus*	Bivalve	5	3	1	[41]
*Avichlamys tellinii*	Bivalve	3	6	1	[20]
*Bakevellia* spp.	Bivalve	4	6	1	[20]
*Bakevellia* cf. *albertii*	Bivalve	4	6	1	[20]
*Bakevellia* cf. *exporrecta*	Bivalve	4	6	1	[20]
*Claraia aurita*	Bivalve	3	4	1	[42]
*Claraia clarai*	Bivalve	3	4	1	[42]
*Claraia stachei*	Bivalve	3	4	1	[42]
*Claraia wangi-griesbachi*	Bivalve	3	4	1	[22]
*Costatoria costata*	Bivalve	5	3	1	[43]
*Eumorphotis* spp.	Bivalve	3	6	1	[44]
*Eumorphotis multiformis*	Bivalve	3	6	1	[44]
*Neoschizodus laevigatus*	Bivalve	5	3	1	[20]
*Neoschizodus ovatus*	Bivalve	5	3	1	[20]
*Scythentolium* sp.	Bivalve	3	5	1	[20]
*Austrotindaria? canalensis*	Bivalve	5	2	3	[36]
*Austrotindaria antiqua*	Bivalve	5	2	3	[36]
*Holocrinus* sp.	Crinoid	2	4	1	[45]
Ophiuroidea	Ophiuroid	3	2	2/5	[46]
cf. *Plagioglypta* sp.	Scaphopod	4	2	3	[47]
*Allocosmia* sp.	Gastropod	3	3	1	[48]
*Coelostylina werfensis*	Gastropod	3	3	1	[47]
*Pseudomurchisonia kokeni*	Gastropod	3	3	1	[47]
*Polygyrina* sp.	Gastropod	3	3	1	[47]
Gastropod sp. A	Gastropod	3	3	1	-
Gastropod sp. B	Gastropod	3	3	1	-
cf. *Worthenia* sp.	Gastropod	3	3	1	-
*Warthia vaceki*	Gastropod	3	2	2	[13]
Bellerophontidae with costae	Gastropod	3	2	2	[13]
*Werfenella rectecostata*	Gastropod	3	2	4	[49]
*Natiria costata*	Gastropod	3	2	4	[20]
Ostracod	Ostracod	3	2	2	-
Microconch	Microconchid	3	6	1	[50]

Diversity was measured using species richness (*S*) and the Simpson Diversity Index (1-D). As the number of individuals varied between samples the Simpson Diversity Index was converted to an effective diversity (Δ [38]), which allows the impact of evenness on richness to be quantified. The Kruskal-Wallis test was used to investigate differences in the median diversity between different units/members and substages. Cluster analysis using an unweighted pair-group average cluster, was applied to recognise those species that tend to co-occur in samples and to group together samples of similar taxonomic composition using the Bray-Curtis similarity matrix. The similarity profile test (SIMPROF) was applied to determine significant differences between the clusters [39] and the similarity percentages routine (SIMPER) was used to determine which species were responsible for the greatest similarity within the groups. Non-metric multi-dimensional scaling ordination methods using a Bray-Curtis similarity matrix were used to visualise patterns in multivariate data (following [12]). A permutational ANOVA (PERMANOVA) was used to test if there were significant changes in the composition of fossil assemblages between units/members, substages and facies. A permutation test of homogeneity of dispersions (PERMDISP) was used to investigate the changes in the dispersion of groups. P-values of <0.05 were used to reject the null hypothesis. Multivariate statistical analysis was performed using PRIMER & PERMANOVA v6.

## Results

### Carbon isotopes

An overall negative isotope excursion from 4.3‰ to -2.6‰, between the upper Bellerophon Formation to the mid-Mazzin Member, is recorded. By the lower Siusi Member isotope values rise to more ‘stable’ values around ~1.3‰. In the mid-Siusi Member carbon isotope values record a gradual rise to ~2.8‰ before a ~2.6‰ negative excursion between the mid- and upper-Siusi Member. In the upper Siusi Member and unit A of the Gastropod Oolite Member, isotope values become more positive and reach peak values for the Werfen Formation of 6.3‰ at the Induan/Olenekian boundary [24]. Unit B of the Gastropod Oolite Member then records a drop from 6.3‰ to -0.5‰. Carbon-isotope values remain negative throughout the Campil Member, becoming more negative up section decreasing to -2.0‰. Although becoming increasingly noisy, the carbon isotopes from the upper Werfen Formation, record a positive isotope excursion near the base of the Val Badia Member, and show an overall decrease through the Spathian (Fig 3).

### Alpha diversity

**Fig 4:**
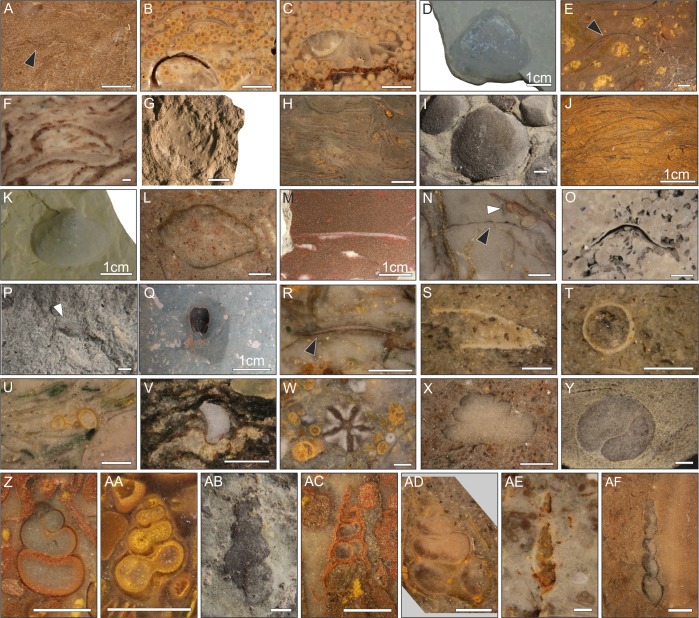
Fossil invertebrates from the Werfen Formation, Dolomites, Italy. **A)** Bivalve sp.A, Mazzin Member; **B)** Bivalve sp.B, Tesero Member; **C)** Bivalve sp.C, Tesero Member; **D-E)** cf. *Unionites danocinus*, Siusi Member; **F)** *Neoschizodus ovatus*, San Lucano Member; **G)** *Claraia clarai*, Mazzin Member; **H)** *Claraia clarai*, Siusi Member; **I-J)** *Claraia wangi-griesbachi*, Mazzin Member; **K)** *Austrotindaria antiqua*, Siusi Member; **L)** *Austrotindaria* spp, Campil Member; **M)** *Scythentolium* sp, Cencenighe Member; **N)** *Eumorphotis* spp. (black arrow) and a microconchid encrusted on a bivalve shell (white arrow), Siusi Member; **O)** *Bakevellia* spp, Cencenighe Member; **P)** *Lingularia yini*, Mazzin Member; **Q)** *Lingularia borealis*, Siusi Member; **R)** *Lingularia* spp, Gastropod Oolite Member; **S-T)** cf. *Plagioglypta* sp, Siusi Member; **U)** Microconchid, Siusi Member; **V)** Ophiuroid ossicle, Siusi Member; **W)** *Holocrinus* sp, Cencenighe Member; **X)** cf. *Worthenia* sp, Campil Member; **Y)** *Natriria costata*, Cencenighe Member; **Z)** *Coelostylina werfensis*, Siusi Member; **AA)** High-spired gastropod sp.B, Siusi Member; **AB-AC)** *Polygyrina* sp, Siusi Member; **AD)** *Pseudomurchisonia kokeni*, Siusi Member; **AE)** *Allocosmia* sp, Cencenighe Member; **AF)** High-spired gastropod sp.A, Siusi Member. Scale bar = 1mm, except D, K, M and Q.

A total of 37 benthic invertebrate species from 29 genera were identified (Table 1; Fig 4), representing bivalves, gastropods, microconchids, ostracods, brachiopods, scaphopods, ophiuroids and crinoids. The MNI ranges from 1 to 867 per sample, and 186 samples have a large enough abundance (i.e. MNI >20) for quantitative analysis (S2 Table).

**Fig 5:**
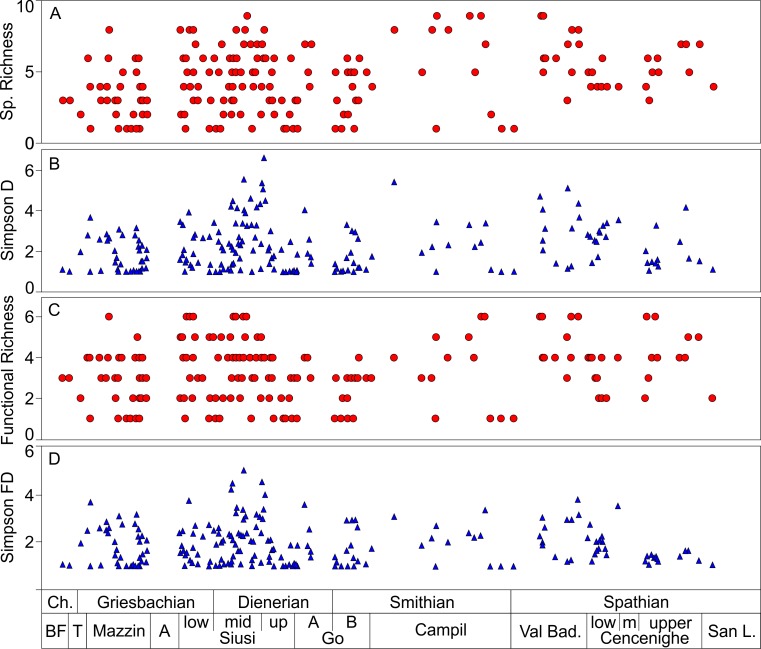
Distribution of taxonomic and functional changes through the Werfen Formation. **A)** Changes in taxonomic richness; **B)** Changes in Simpson Diversity; **C)** Changes in functional richness; **D)** Changes in Simpson functional diversity. Stratigraphic framework as in Fig 2. Ch.—Changhsingian, BF—Bellerophon Formation, T–Tesero, A–Andraz, GO—Gastropod Oolite, Val bad.–Val Badia, San L.–San Lucano.

**Fig 6:**
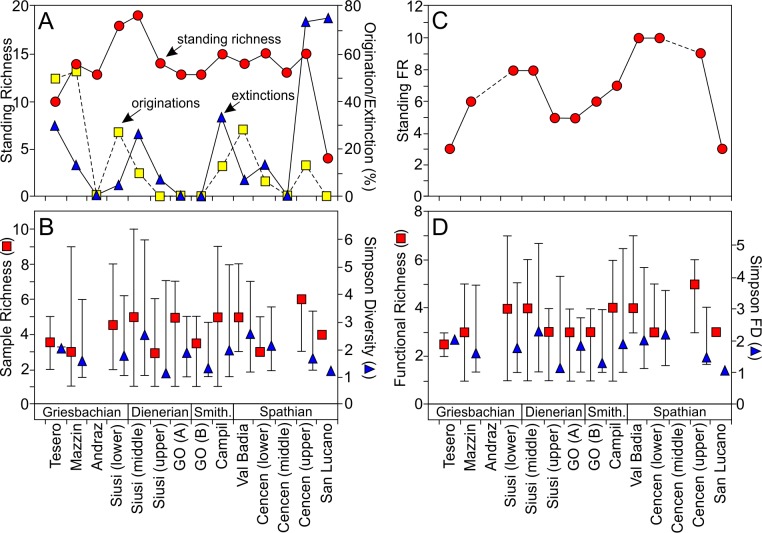
Alpha diversity dynamics through the Werfen Formation. **A)** Changes in taxonomic richness: standing richness (circles), originations (squares) and extinctions (triangles). **B)** Changes in median sample richness (squares) and Simpson Diversity (triangles), ranges are shown by the vertical lines. **C)** Number of modes of life recorded in each unit of the Werfen formation. Standing FR–Standing Functional Richness. **D)** Changes in sample functional richness (squares) and Simpson Functional Diversity (triangles), ranges are shown by the vertical lines. Simpson FD–Simpson Functional Diversity.

Taxonomic richness does not increase with time but fluctuates across the Werfen Formation, with the peritidal units (i.e. Andraz; Gastropod Oolite (Unit A), mid-Cencenighe and San Lucano members) either comprising only a few samples or gaps in the shelly fossil record (Figs 3 and 5). The Tesero Member records a low standing richness, which is due to the impact of the late Permian mass extinction, limited range of facies and edge effects. The overlying Mazzin Member is relatively diverse, with 14 taxa recorded. Excluding the Tesero, Siusi and San Lucano members, standing diversity in the Mazzin Member is comparable to the rest of the Werfen Formation (Fig 6A). Additionally, the highest origination rates are recorded in the Mazzin Member (54%; Fig 6A) associated with the appearance of ophiuroid ossicles and the first regional appearances of *Lingularia yini*, *Claraia wangi-griesbachi*, *C*. *clarai*, *Polygyrina* sp., *Neoschizodus laevigatus*, *Austrotindaria? canalensis*, *Austrotindaria antiqua* and *Eumorphotis* spp. and therefore, the high origination rates are not due to edge effects. Median sample richness, however, is significantly lower than most the sampled units of the Werfen Formation (Fig 6B; S3 Table). Sample richness is significantly greater (*p* = 0.01; S3 Table) in the overlying lower- and mid-Siusi Member where they reach peak values for the entire Werfen Formation (Fig 6B). This increase is associated with elevated origination and low extinction rates (Fig 6A), and a return to carbon isotope values of 2‰ and a flatter isotope curve (Fig 3). These changes between the Mazzin and lower Siusi members occur despite a similar range of water depths and comparable lithofacies, i.e. distal mid-ramp, and are, therefore, not attributed to a facies bias.

The negative isotope excursion between the mid- and upper Siusi Member (Fig 3), coincides with elevated extinction rates (28%; Fig 6A), with the disappearance of cf. *Unionites donacinus*, *Claraia clarai*, *C*. *stachei*, *C*. *aurita* and bellerophontids. In addition, species richness and Simpson Diversity significantly decline (*p*<0.01 and *p* = 0.05, respectively; S3 Table). Local origination and extinction rates are low throughout the Gastropod Oolite Member (Fig 6A), which encompasses the peak of the positive C-isotope excursion (Fig 3). The Campil Member records two new originations and a corresponding small increase in standing diversity compared to the Gastropod Oolite Member. Sample richness also significantly increases from the unit B of the Gastropod Oolite Member (*p* = 0.03; S3 Table; Fig 6B). The Campil Member records increased extinctions (29%) with three of the four species disappearing being small gastropods (typically <1.5cm): *Polygyrina* sp., cf. *Worthenia* sp. and Gastropod sp. A.

The positive isotope excursion at the Campil/Val Badia member boundary, i.e. SSB, is not associated with significant difference in sample richness or Simpson Diversity (*p* = 0.76 and *p* = 0.83, respectively; S3 Table) or standing diversity. The Val Badia Member records elevated origination rates (29%) after the SSB (Fig 6A). New taxa recorded in this member include the notably larger gastropod species *Natiria costata* and *Werfenella rectecostata* (typically >1.5cm), as well as the crinoid *Holocrinus* sp. which makes its first regional appearance. The overlying Cencenighe Member records the local origination of two bivalves and a gastropod species and in the upper part of that member there is a significant increase in sample richness (*p*<0.01) and origination rates (Fig 6B and 6D).

### Functional diversity

Thirteen different modes of life were recognised in this study (Table 1). Functional richness increases through the lower Werfen Formation, reaching a mid-Siusi Member peak of eight modes of life (Fig 6C), of which a maximum of seven are recorded in any one sample (Figs 5C and 6D). The lower and mid-Siusi Member are significantly more functionally rich than either the Mazzin or Tesero members (Fig 6D; *p =* 0.01, S3 Table). The upper Siusi Member records a significant decline in standing functional richness and in the functional richness of individual samples (*p*<0.01; S3 Table) with the loss of three modes of life (i.e., epifaunal, facultative motile, attached, suspension feeders; epifaunal, slow-moving, surface deposit feeders; and semi-infaunal, slow-moving, miners), associated with the disappearance of *Claraia*, bellerophontids and scaphopods, respectively.

Functional richness of individual samples remains low through the Gastropod Oolite Member, and functionally diverse samples are not recorded again until the Campil Member, associated with the reappearance of ophiuroids and the first records of semi-infaunal, stationary attached, suspension feeders (e.g. *Bakevellia*) in this study. A major increase in overall functional richness occurs between the Campil and Val Badia members (Fig 6C), associated with the first records of erect, facultatively motile, suspension feeders (i.e., crinoids) and epifaunal, slow-moving, grazers (e.g., *Werfenella rectecostata*), but there is no significant change (*p* = 0.36; S3 Table) in the median richness of individual samples (Fig 6D). Overall functional diversity remains high through most of the Spathian until the uppermost Werfen Formation, with median sample richness reaching a maximum in the upper Cencenighe Member (Fig 6D). No significant changes are recorded in the Simpson functional diversity between the members of the Werfen Formation (*p =* 0.14), suggesting that differences in functional richness are controlled by rare taxa.

### Changes in taxonomic and functional compositions

**Fig 7:**
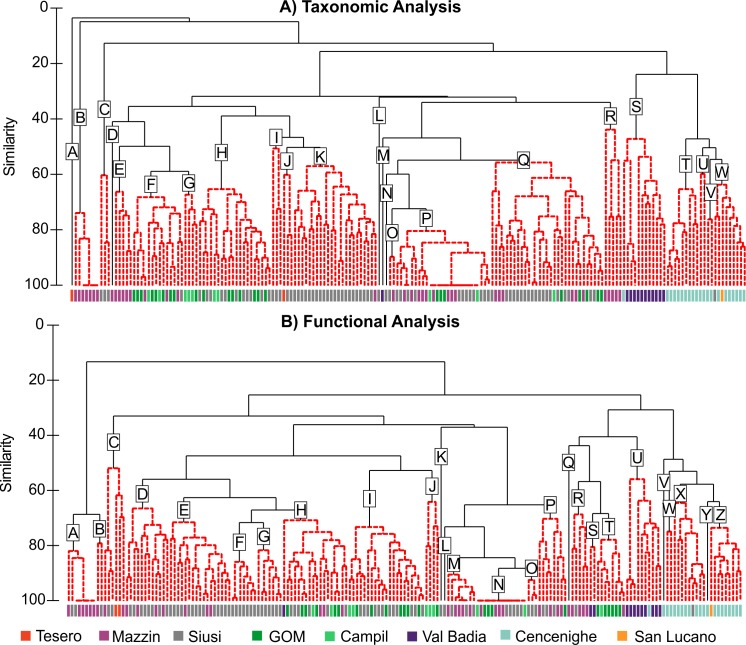
Cluster analysis of the samples from Werfen Formation. **A)** The SIMPROF test identified 23 groups of samples which are statistically distinct (dashed lines; A-W). The groups are interpreted as different benthic biofacies. **B)** The SIMPROF test identified 26 groups of samples which are statistically distinct (dashed lines; A-Z). The groups are interpreted as different benthic ecofacies.

Cluster analysis of samples based on their taxonomic compositions reveals five broad groups at low similarity (<20%) that are divided into 23 quantitative biofacies recognised by the SIMPROF analyses (Fig 7A; S4 Table). The first group comprises a single sample from the Tesero Member that is dominated by Bivalve sp. A and *Warthia vaceki* (Biofacies A). The second group is composed of samples dominated by *Claraia aurita* group (Biofacies B) and is restricted to the Mazzin Member. The third group comprises samples from the Siusi Member that are attributed to a single biofacies (C) which is dominated by *Claraia clarai*. The fourth group includes samples from the Tesero, Mazzin, Siusi, Gastropod Oolite and Campil members, as well as a single sample from the Val Badia Member. Sixteen distinct biofacies can be recognised in this group, characterised by *Lingularia* and microconchids (Biofacies D); microconchids (Biofacies E); *Coelostylina werfensis* and microconchids (Biofacies F, G); *Coelostylina werfensis* (Biofacies H, K); *Austrotindaria* and *Claraia clarai* (Biofacies I); *Warthia vaceki* (Biofacies J); *Plagioglypta* sp. (Biofacies L); *Austrotindaria* (Biofacies M-Q) and ostracods; and *Austrotindaria* and microconchids (Biofacies R). The high similarity between the biofacies is due to the high abundance of *Austrotindaria*, *Coelostylina* and microconchids (S4 Table). Group 5 comprises almost all of the Spathian samples and a single Siusi Member sample and divides into five biofacies dominated by: *Natiria costata*, microconchids and *Eumorphotis* (Biofacies S); *Eumorphotis* and *Neoschizodus* spp. (Biofacies T); *Neoschizodus* spp. and *Bakevellia* spp. (Biofacies U); *Neoschizodus* spp. and *Scythentolium* sp. (Biofacies V); and *Neoschizodus* spp. (Biofacies W).

In contrast, cluster analysis of samples based on their functional composition reveals 26 quantitative ecofacies that can be discriminated by a SIMPROF test (Fig 7B; S5 Table), with an increased similarity between the Spathian and pre-Spathian samples due to the dominance of the epifaunal, stationary, attached, suspension feeders. Except for three samples from the Val Badia Member, all of the Spathian samples cluster as a separate group (Ecofacies U-Z) that are dominated by: shallow-infaunal, facultatively motile, unattached, suspension feeders (Ecofacies W, Y, Z); semi-infaunal, stationary, attached, suspension feeders (Ecofacies Q); epifaunal, stationary, attached, suspension feeders (Ecofacies X); and epifaunal, slow-moving, grazers (Ecofacies U). The three remaining Spathian samples are dominated by shallow infaunal, slow-moving, miners (Ecofacies H) and epifaunal, stationary, attached, suspension feeders (Ecofacies S). Ecofacies A-G, K-M and O are restricted to both the Griesbachian and Dienerian, and are dominated by epifaunal, facultatively motile, attached, suspension-feeders (A, B, D); epifaunal, slow-moving deposit-feeders (C); epifaunal, facultatively motile, unattached, suspension-feeders (E, G); shallow infaunal, slow-moving, miners (F, L-O); and semi-infaunal, slow-moving, miners (K). The remaining pre-Spathian samples are dominated by epifaunal, facultatively motile, unattached, suspension-feeders (Ecofacies I-J) and shallow infaunal, slow-moving, miners (Ecofacies N-P). None of the identified ecofacies are restricted to the Smithian.

**Fig 8:**
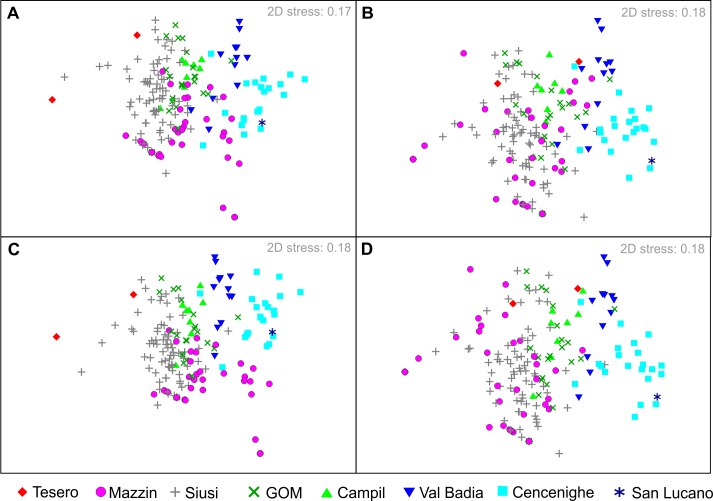
Non-metric multi-dimensional scaling (nMDS) ordination of samples, grouped according to members of the Werfen Formation. **A)** Taxonomic composition of samples using MNI. **B)** Functional composition using MNI. **C)** Taxonomic composition of samples using the number of bioclasts. **D)** Functional composition of samples using bioclasts.

The nMDS plots have stress values of 0.17 and 0.18 which suggests that they are a good representation of the data (Fig 8). Samples from the Tesero, Mazzin, Siusi, Gastropod Oolite and Campil members largely overlap in the centre of the ordinations because of the common dominance of *Austrotindaria*, *Coelostylina* and microconchids, and their associated modes of life, in these Griesbachian, Dienerian and Smithian assemblages (Fig 8A and 8B). In contrast, the samples from the other (i.e., Spathian) members of the Werfen Formation plot in a different part of the ordinations (Fig 8A and 8B). The PERMANOVA results show that significant differences occur between the centroids of all members of the Werfen Formation, except between the Gastropod Oolite and Campil members (Table 2).

**Table 2:** PERMANOVA results testing the difference between the centroids of the composition of samples for the members of the Werfen Formation. A) Main Test. P(Perm) = permutational *p*-value. P(MC) = Monte Carlo *p*-value. B) Pair-wise comparisons. Comparisons between the different members based on taxonomic composition are shown in **bold** and functional composition is shown in *italics*. The Tesero and San Lucano members were excluded from the tests as they have <3 samples. GOM = Gastropod Oolite Member.

**A)**	**df**	**SS**	**MS**	**Pseudo-F**	**P(perm)**	**P(MC)**
**Taxonomic**	7	180450	25779	14.377	0.001	0.001
**Functional**	7	146850	20979	13.318	0.001	0.001
**B)**	**Mazzin**	**Siusi**	**GOM**	**Campil**	**Val Badia**	**Cencenighe**
**Mazzin**		*<0*.*01*	*<0*.*01*	*<0*.*01*	*<0*.*01*	*<0*.*01*
**Siusi**	**<0.01**		*<0*.*01*	*0*.*02*	*<0*.*01*	*<0*.*01*
**GOM**	**<0.01**	**<0.01**		*0*.*79*	*<0*.*01*	*<0*.*01*
**Campil**	**<0.01**	**0.01**	**0.711**		*<0*.*01*	*<0*.*01*
**Val Badia**	**<0.01**	**<0.01**	**<0.01**	**<0.01**		*<0*.*01*
**Cencenighe**	**<0.01**	**<0.01**	**<0.01**	**<0.01**	**<0.01**	

### Sample dispersion

**Fig 9:**
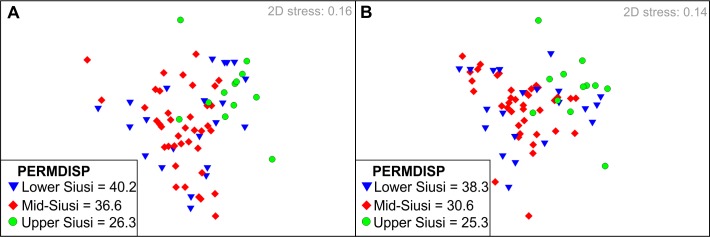
nMDS ordination showing the compositional changes between the different units of the Siusi Member. **A)** Taxonomic compositions. **B)** Functional compositions.

The Tesero and San Lucano members have too few samples, (<3), for a comparison of sample dispersion. The PERMDISP results show that the dispersion of samples is significantly different between the remaining members of the Werfen Formation both taxonomically (*p* = 0.05) and functionally (*p =* 0.01), with the Mazzin Member having the largest dispersion (Table 3). PERMDISP analyses of the different units of the Siusi Member, which records elevated extinctions (Fig 9A), also shows a decrease in taxonomic and ecological sample dispersion between the mid- and upper Siusi Member (*p<*0.01 and *p =* 0.03, respectively; Fig 9). This decline in dispersion is due to the disappearance of those taxa that previously dominated biofacies (e.g. *Claraia* and *Warthia*). In contrast, although PERMDISP decreases between the lower and mid-Siusi Member, i.e. through the G/D boundary, it is not taxonomically or ecologically significant (*p =* 0.27; *p =* 0.15, respectively).

**Table 3:** PERMDISP results of the composition of samples between the different members of the Werfen Formation. The Tesero and San Lucano members were excluded from the tests as they have <3 samples.

		Taxonomic Analysis		Functional Analysis	
Member	Size	Average	SE	Average	SE
Mazzin	38	45.7	2.2	43.8	2.3
Siusi	77	39.3	1.5	35.4	1.7
GOM	24	36.2	3.5	34.2	3.7
Campil	10	36.8	5.0	33.6	6.3
Val Badia	12	32	3.4	30.4	3.5
Cencenighe	21	34.2	3.2	28.4	3.0

### Importance of depositional setting

**Fig 10:**
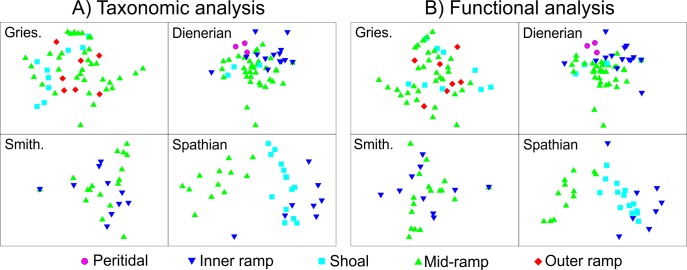
nMDS ordination of the composition of samples grouped according to sedimentary facies within each substage. **A)** Taxonomic analysis. **B)** Functional analysis. Gries–Griesbachian, Smith.–Smithian.

A two-way PERMANOVA between substage and sedimentary facies shows that both substage and depositional environment significantly affect the taxonomic and functional composition of samples in the nMDS ordination (*p =* 0.01 and *p =* 0.01, respectively). Ordination shows, however, that within the Griesbachian, Dienerian and Smithian substages samples from different depositional settings overlap and are not well-differentiated from each other taxonomically or ecologically (Fig 10). In contrast, the Spathian samples from the different environments are strongly differentiated and the ordination recovers an environmental gradient with samples from the mid-ramp facies plotting on the left through to those from the inner ramp on the right (Fig 10).

### Echinoderm-dominated biofacies

If the total number of specimens per sample is used to reconstruct relative abundances rather than MNI, using the same samples, the ordination of samples shows similar relationships and amount of dispersion (Fig 8C and 8D). The quantitative biofacies also remain unchanged for the most part with 23 out of the 26 biofacies having comparable compositions (S6 Table). The differences in the remaining biofacies are associated with the increased dominance of echinoderms. Echinoderm-dominated biofacies were not recognised using the MNI approach, whereas, using the number of bioclasts three of the biofacies are dominated by echinoderms. The increased abundance of ophiuroids (biofacies H-I; S6 Table) and associated mode of life also mean that the Val Badia and Cencenighe members more closely resemble the Siusi, Gastropod Oolite and Campil members (Fig 8C and 8D). The increased abundance of *Holocrinus* sp. (Biofacies F; S6 Table) is restricted to the Spathian.

## Discussion

### Initial recovery

Based on palaeoecological parameters such as tiering and bioturbation, early studies of the Werfen Formation recorded initial signs of benthic recovery in the lower-mid Siusi Member with a measurable advance at the G/D boundary [5,16]. Despite differences in methodology and analyses, and a significant increase in sampling resolution, the present study also records measurable increases in the taxonomic and functional Simpson diversities of samples in the mid-Siusi Member (Figs 5 and 6), which supports the view that the G/D boundary marks a major advance in recovery of benthic ecosystems in northern Italy. Prior to that, a significant rise in alpha diversity is also recorded between the Mazzin and lower Siusi members, with increases in the median taxonomic and functional richness of individual samples, as well as a significant rise in standing diversity (Fig 6A). Additional evidence of benthic recovery between the Mazzin and lower Siusi members includes significant body size increases in bivalves, gastropods and brachiopods [51]; significant burrow diameter increase [16,52]; and an increase in ichnodiversity and infaunal tiering, including local reappearance of the key ichnogenus *Thalassinoides* [4,16,53] (S1–S6 Figs). This recovery signal also coincides with a more ‘stable’ carbon isotope curve around 2‰ (Fig 3), as is the recovery of marine communities in the Middle Triassic [9].

The Mazzin and lower Siusi members were deposited at similar water depths, i.e. distal mid-ramp, and so this recovery signal occurs in the absence of significant facies change. The diverse assemblages recorded in these members are mostly restricted to thin beds in an otherwise anoxic/dysoxic setting [54]. This implies that diverse benthic communities were only able to colonize distal mid-ramp settings during intervals of slightly elevated oxygen concentrations, and/or that these assemblages were transported offshore during storms and that initial stages of recovery were restricted to settings aerated by wave activity, i.e. the habitable zone [55]. Similar depositional settings in the Mazzin and lower Siusi members may explain why the shelly macroinvertebrate assemblages record only very limited compositional change through the late Griesbachian recovery interval. Our analyses and those of [11] demonstrate that the taxonomic and ecological compositions of Mazzin Member samples are very similar to those of the Siusi Member (Figs 7 and 8). Significant shifts in the composition of benthic communities are only recorded later, in the advanced stages of recovery during the Spathian (Figs 7 and 8). Even though the habitable zone hypothesis, i.e. oxygenation of the seafloor via wave aeration [55], explains a survival mechanism, the limited ecological complexity shows that the habitable zone must have still been stressed by other environmental factors, including those associated with increased runoff (e.g. [12]) and low levels of atmospheric oxygen (e.g. [56]).

The macrofauna of the Mazzin and Siusi members are also often characterized as eurytopic (e.g. [7]) and the late Griesbachian recovery interval did not lead to the reappearance of specialist invertebrate groups, such as crinoids or articulate brachiopods. These groups are not recorded in northern Italy or in other western Palaeotethys localities until the Spathian [12,16]. Elsewhere, such as in Oman, late Griesbachian shelly assemblages record much greater ecological complexity, which led [5] to conclude that rates of recovery varied between regions. Despite significant additional sampling in the past decade, including this present study, it is clear that the Mazzin and Siusi members record no representatives of the erect tier, no articulate brachiopods, have half the diversity of equivalent age strata from Oman, and contain smaller gastropods (cf. [5,57–58]). Late Griesbachian communities from northern Italy were less diverse taxonomically and functionally and at a less advanced state of recovery than those from Oman, supporting the view that the pace and magnitude of recovery varied between different regions[5,14].

The Griesbachian Oman assemblages are, however, partially silicified [5] and so some of these differences might be attributable to differences in fossil preservation, e.g. the taxonomic richness of originally aragonitic molluscs. The absence of calcitic crinoids and brachiopods in the lower Werfen Formation, however, and differences in key traits such as body size, instead suggest that the recorded differences in ecological complexity between the two regions are real and not the product of rock record bias. Furthermore, there is no globally synchronous initial recovery in the late Griesbachian, as previously suggested [4], as the initial recovery in the Dolomites occurs at least one conodont zone after initial recovery in Neotethys [5] and two conodont zones after initial recovery in the Boreal Ocean [36,55].

### Dienerian crisis

Although there is no isotope excursion associated with the G/D boundary in the Dolomites, a trend towards more positive values begins in the mid-Siusi Member, associated with an increase in taxonomic and functional Simpson diversities (Fig 6B) and standing taxonomic richness (Fig 6A). As noted previously [11], however, and as recorded in neighbouring regions such as Hungary [12], there is no significant change in the composition of benthic faunas (Fig 8). The trend towards more positive C-isotope values is temporarily interrupted at the base of the upper Siusi Member by a ~2‰ negative isotope excursion, before resuming through the remaining Siusi Member (Fig 3). This brief negative isotope excursion coincides with a reduction of taxonomic and functional diversity of samples (Figs 5B, 5C and 6B–6D) and the upper Siusi member records a sharp reduction in standing diversity, the last appearances of some eurytopic taxa (e.g. *Claraia* and *Warthia* [24]), and a significant reduction in the heterogeneity of sample composition (Fig 9). A benthic crisis during the Dienerian has previously been recognized in the western US and Dolomites [10–11,15]. In the Dolomites, the extinctions are associated with enriched δ^34^S and depleted δ^13^C values, interpreted by [59] as indicative of very sluggish ocean circulation. In addition, in the Neotethys an expansion of the oxygen minimum zone into shallower settings is recorded in the upper Dienerian [60]. These extinctions in the shelly faunal record, however, are not attributed to a facies change as comparable lithologies and water depths are recorded in Unit B of the Gastropod Oolite Member.

The Gastropod Oolite Member records no new taxa and no last appearances, but it does record a slight increase in burrow size and the number of ichnotaxa [16,53]. In addition, the composition of assemblages is comparable to those of the Griesbachian and Dienerian. Subsequent environmental changes (indicated by a positive isotope excursion; Fig 3) across the Dienerian/Smithian boundary, therefore, did not cause a major disruption to the benthic invertebrate community. The lack of turnover associated with the Dienerian crisis is likely because the taxa that occur and dominate assemblages from the Mazzin, Siusi and Gastropod Oolite members are characterized as eurytopic and so, the lack of endemism and complex specialists prior to the Smithian may explain why there is no significant shift in the composition of the benthos.

### Smithian/Spathian event

Continuous warming during the Smithian [8] and/or changes in the topography of the hinterland caused a regional increase in humidity, weathering and runoff which lead to increased siliciclastic loads delivered to the seafloor throughout western Palaeotethys [12,16] and possibly elsewhere [15]. The Campil Member records elevated extinction rates (33%; Fig 6A), and in the fine-grained siliciclastic facies the benthic assemblages have low diversities and are dominated by *Austrotindaria* (= *Unionites*) [11,15] (Fig 7). These data support previous suggestions (e.g. [16]) that factors associated with increased runoff caused a reversal in recovery during the Smithian.

The coarser lithologies and carbonate-rich beds from the Campil Member, however, record relatively high species richness (*S* = 9) and a significant increase in sample richness (*p*<0.01; Fig 6B). Griesbachian, Dienerian, and Smithian samples overlap in the nMDS ordination (Fig 8) showing that the taxonomic and functional composition of the assemblages is comparable and suggesting that the Campil Member does not record a major phase shift for benthic ecosystems. The Campil Member also records an increase in origination rates (Fig 6A) and the appearance of two modes of life (semi-infaunal, facultatively motile, attached suspension-feeders and epifaunal, facultatively motile, unattached suspension-feeders) associated with the immigration of bivalve taxa [7]. Furthermore, the size of bivalves increases into the Campil Member [51]. Therefore, even though the Campil Member records a more stressful (e.g. estuarine) facies and a restriction of diverse benthic assemblages it does not record an overall biotic crisis.

The positive carbon isotope excursion at the SSB (Fig 3) is associated with elevated extinction rates (28%) in the Smithian Campil Member, however, because elevated origination rates are also recorded (29%) in the Val Badia Member there is no significant difference in sample or standing diversity (Fig 6A). Major environmental changes are associated with the elevated extinction and subsequent origination rates across the SSB that have been recorded globally. A peak in mercury concentrations in the upper Smithian of Svalbard has been interpreted as indicating that subsequent Siberian Traps volcanism drove the warming that may have led to the extinctions [61]. In addition, in the Neotethys and the eastern Palaeotethys the oxygen minimum zone appears to have expanded into shallow settings in the upper Smithian, before retreating in the lower Spathian with global cooling and amelioration of extreme climate conditions, prompting a Spathian diversification event [60,62–63].

Spathian assemblages are significantly different taxonomically and functionally from pre-Spathian ones (Figs 7 and 8), and the SSB therefore marks a major shift in the composition of the benthic communities. A similar shift in taxonomic composition across the SSB has been recorded in the western US and attributed to a greater relative abundance of previously rare taxa [10]. In the Dolomites, however, the changes are better attributed to taxonomic turnover as well as changes in the relative abundances of existing taxa. Extinctions resulted in a decrease in the relative abundance of small gastropods (e.g. *Polygyrina* sp. and cf. *Worthenia* sp.) and the infaunal deposit-feeding bivalve *Austrotindaria*, whilst the newly evolved, larger gastropod taxa, e.g. *Natiria costata* and *Werfenella rectecostata*, and the infaunal suspension-feeding bivalve *Neoschizodus ovatus* dominate assemblages for the first time (Figs 7 and 8). A similar functional turnover can also be recognized between the Bódvaszilas Sandstone and Szin Marl formations (i.e. SSB in Hungary [12]), which also records deposition in western Palaeotethys. This significant taxonomic and functional shift is also due in part to the reassignment of the facultatively motile, suspension feeder *“Unionites”* to the motile, deposit-feeding *Austrotindaria* (following the identification of the first silicified fauna from the Early Triassic;[36]) which was not recognised in previous studies (e.g. [11–12]) in the western Palaeotethys.

The SSB in the Werfen Formation is also marked by major facies change that may, at least in part, explain the significant palaeoecological changes, although the Spathian strata do record comparable facies and water depths to some of the pre-Spathian members. Linear sedimentation rates decrease markedly in the Spathian (Table 4) and may provide an explanation for an ecological shift from infaunal deposit-feeding to infaunal suspension-feeding bivalves and the appearance of larger grazing gastropods. Stenohaline taxa such as *Holocrinus* sp., *Neoschizodus ovatus* and *Natiria costata*, appear and dominate assemblages for the first time, suggesting more normal marine salinities in the lower Spathian [16,24,47]. Although crinoid biofacies were not recognised using the MNI approach, bioclast analysis suggests that the crinoid *Holocrinus* sp. was a major component of some Spathian benthic communities. Finally, it has long been noted that the Spathian Val Badia Member records the first appearance of ammonoids in the Werfen Formation (e.g. [20,28]), which has been interpreted as reflecting improved connectivity to the open ocean further east due to tectonic changes at that time (e.g.[64]). Therefore, even though environmental stress may not have been persistent prior to the Spathian, the rapidity of environmental changes indicated by the carbon isotope record appears to have had limited the recovery of certain taxa and functional groups. Alternatively, a combination of synsedimentary tectonism and sea-level rise, known to have affected deposition of the Werfen Formation (e.g. [65]), may have provided a better connection to fully marine conditions allowing for the recovery of stenohaline taxa.

**Table 4:** Changes in the linear sedimentation rate during deposition of the Werfen Formation. Substage durations after [66–69].

Stage/ substage	Thickness (m)	Duration (m.y)	Sedimentation (m/m.y)
**Siusi Section**			
Induan	146	0.9 ±0.4	162
**l’Uomo and Costabella sections**			
Induan	199	0.9 ±0.4	221
Smithian	146	0.6 ±0.6	234
**Val Averta section**			
Spathian	157	3.4 ±0.5	46

The Spathian members of the Werfen Formation record significant benthic recovery, as evidenced by increased functional diversity (Fig 6B), establishment of an environmental gradient in faunal compositions, i.e. increase in β-diversity (Fig 10), expansion of infaunal and erect tiering, widespread bioturbation, and an increase in burrow diameters[16]. This Spathian recovery signal coincides with the amelioration of extreme hothouse conditions [8] and reduced sedimentation rates [70] (Table 4). The Spathian may, therefore, have also been associated with an increased latitudinal temperature gradient allowing improved ocean circulation [59,63], and less detrimental environmental conditions allowing a shift to a more ‘advanced’ recovery state. Environmental changes around the SSB may, therefore, have initially been detrimental causing extinctions but then beneficial in the Spathian as the extreme conditions quickly ameliorated and the benthos rapidly diversified into newly available ecospace.

Hausmann and Nützel [71] demonstrated that the α- and β-diversity of Middle and Late Triassic assemblages were significantly higher than those of the Spathian. The Spathian assemblages from the Werfen Formation also lack some benthic groups that dominated assemblages in the Changhsingian and Middle Triassic such as dasycladaceans, sponges, or articulate brachiopods (e.g. [72–74]). Furthermore, the maximum size of the benthos, the gastropods, bivalves, crinoids, and infauna, are noticeably smaller than in the Middle Triassic and prior to the late Permian mass extinction event [6,9]. The ‘advanced’ recovery state in the Spathian of the western Palaeotethys does not, therefore, represent an equivalent level of ecological complexity as recorded either prior to the mass extinction event or in the Middle Triassic.

## Conclusions

High-resolution sampling and quantitative analysis of fossil assemblages spanning the entire Lower Triassic of northern Italy show that benthic marine ecosystems underwent their first significant recovery in the late Griesbachian. During the Dienerian, extinctions led to a reduction in functional and taxonomic diversity and reduced sample heterogeneity, which apparently curtailed recovery. Benthic communities recovered somewhat during the late Dienerian and Smithian before further extinctions near the Smithian/Spathian boundary. The early Spathian is associated with a positive shift in C-isotopes and marks a significant change in the composition of benthic faunas to a more diverse, functionally rich community, the return of stenohaline taxa, habitat differentiation and the establishment of an environmental gradient, and the occupation of erect and deep-infaunal tiers. This second major recovery phase resulted in levels of ecological complexity that were not recorded prior to the Spathian in northern Italy, and may have been due to the relaxation of environmental stresses that had previously limited recovery of the benthos.

## Supporting information

S1 Fig Stratigraphic section, position of samples (arrows) and ichnofabric indices (ii) at the Siusi section. Lithostratigraphy follows [24]. The occurrence of *H*. *parvus* (after [59]) marks the Permian/Triassic boundary. LPE = late Permian extinction. The position of the late Permian mass extinction is interpreted from the nearby Bulla section after [33]. OU–Ostracod Unit. BM = Bulla Member. Colour in the lithology column refers to the rock colour observed in the field.
(TIF)

S2 Fig Stratigraphic section, position of samples (arrows) and ichnofabric indices (ii) at the Tesero section. Lithostratigraphy after [24]. BF = Bellerophon Formation. OU = Ostracod Unit. B = Bulla Member. For key see S1 Fig.
(TIF)

S3 Fig Stratigraphic section, position of samples (arrows) and ichnofabric indices (ii) at the Rio di Pantl section. Lithostratigraphy after [24]. For key see S1 Fig.
(TIF)

S4 Fig Stratigraphic section, position of samples (arrows) and ichnofabric indices (ii) at the l’Uomo section. Lithostratigraphy after [24]. For key see S1 Fig.
(TIF)

S5 Fig Stratigraphic section, position of samples (arrows) and ichnofabric indices (ii) at the Costabella section. Lithostratigraphy after [24]. For key see S1 Fig.
(TIF)

S6 Fig Stratigraphic section, position of samples (arrows) and ichnofabric indices (ii) at the Val Averta section. Lithostratigraphy after [24]. For key see S1 Fig.
(TIF)

S1 Table Sedimentary facies and depositional environments. (PDF)

S2 Table Dolomites Raw Data (Minimum number of individuals). (XLSX)

S3 Table Kruskal-Wallis pairwise comparisons. (XLSX)

S4 Table MNI Biofacies. (XLSX)

S5 Table MNI Ecofacies. (XLSX)

S6 Table Bioclasts Biofacies. (XLSX)

S1 Text Supporting Information for Fig 3. (PDF)

S2 Text Polished Slab Taxonomic Sheet. (PDF)
