# Supplementary figures and images for: Subsequent biotic crises delayed marine recovery following the late Permian mass extinction event in northern Italy
Source: PLoS One. 2017 Mar 15;12(3):e0172321. doi: 10.1371/journal.pone.0172321 (PMC5351997; doi:10.1371/journal.pone.0172321)

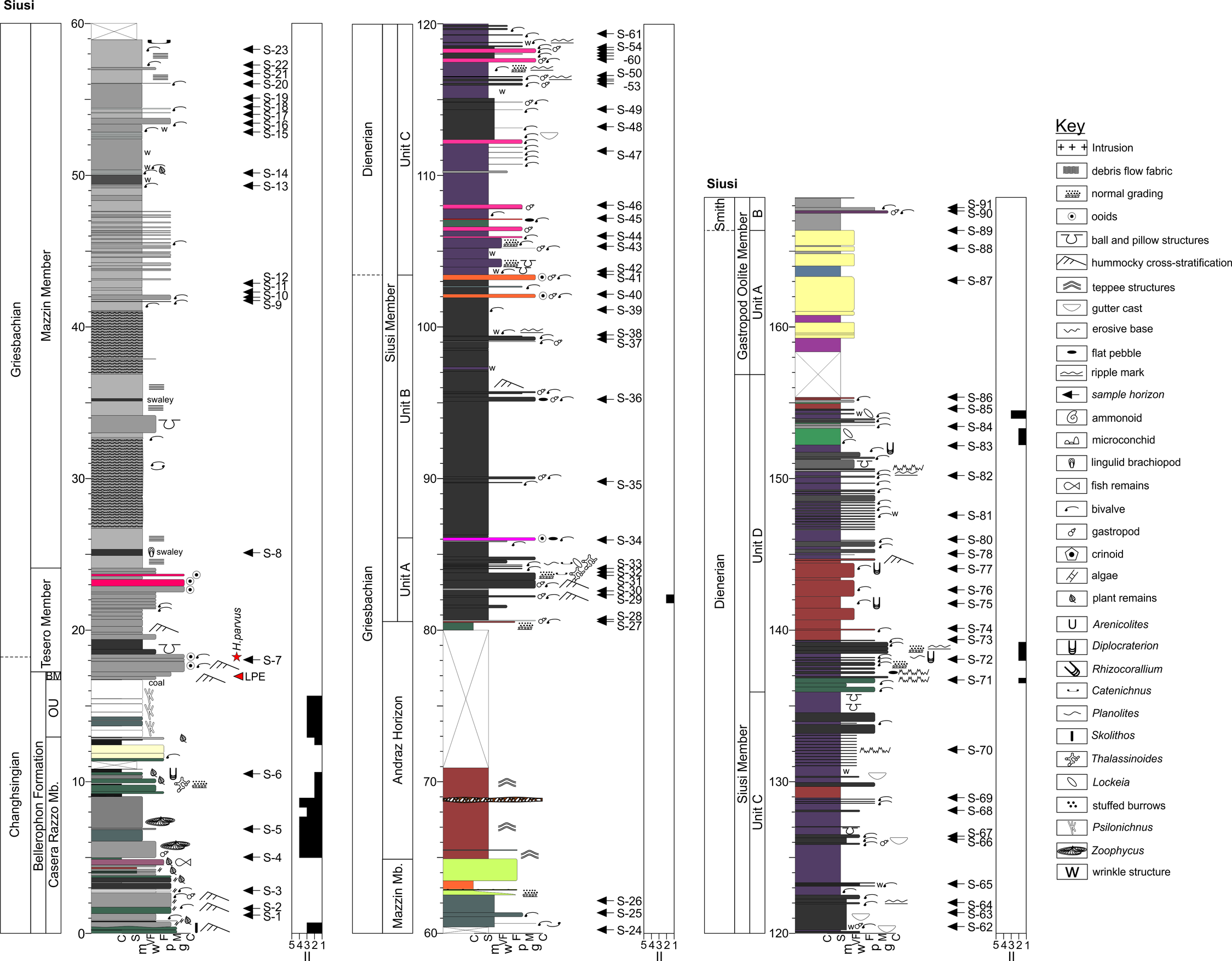

Supplement: S1 Fig — Lithostratigraphy follows [24]. The occurrence of H. parvus (after [59]) marks the Permian/Triassic boundary. LPE = late Permian extinction. The position of the late Permian mass extinction is interpreted from the nearby Bulla section after [33]. OU–Ostracod Unit. BM = Bulla Member. Colour in the lithology column refers to the rock colour observed in the field. (TIF) [file pone.0172321.s001.tif]

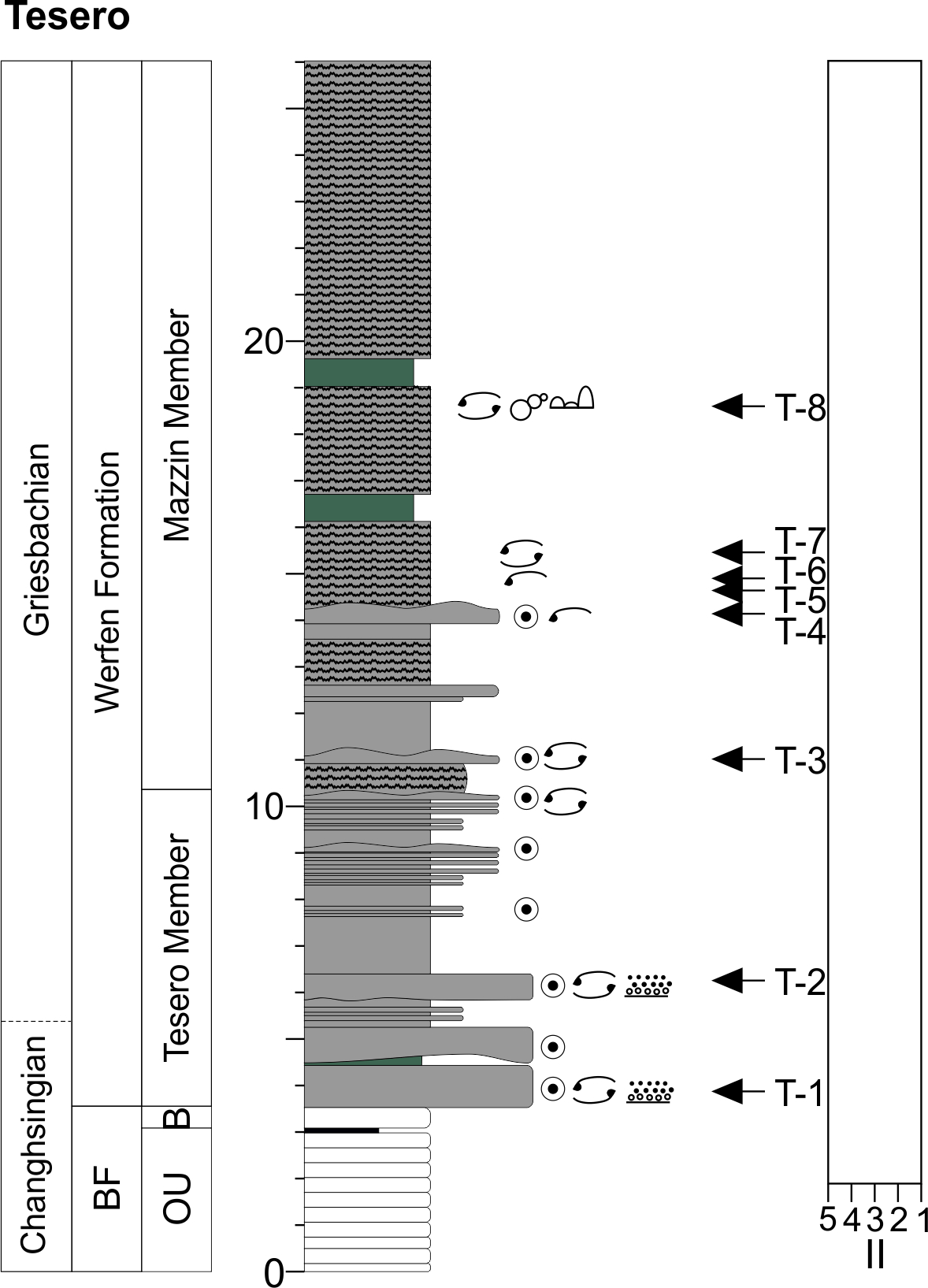

Supplement: S2 Fig — Lithostratigraphy after [24]. BF = Bellerophon Formation. OU = Ostracod Unit. B = Bulla Member. For key see S1 Fig. (TIF) [file pone.0172321.s002.tif]

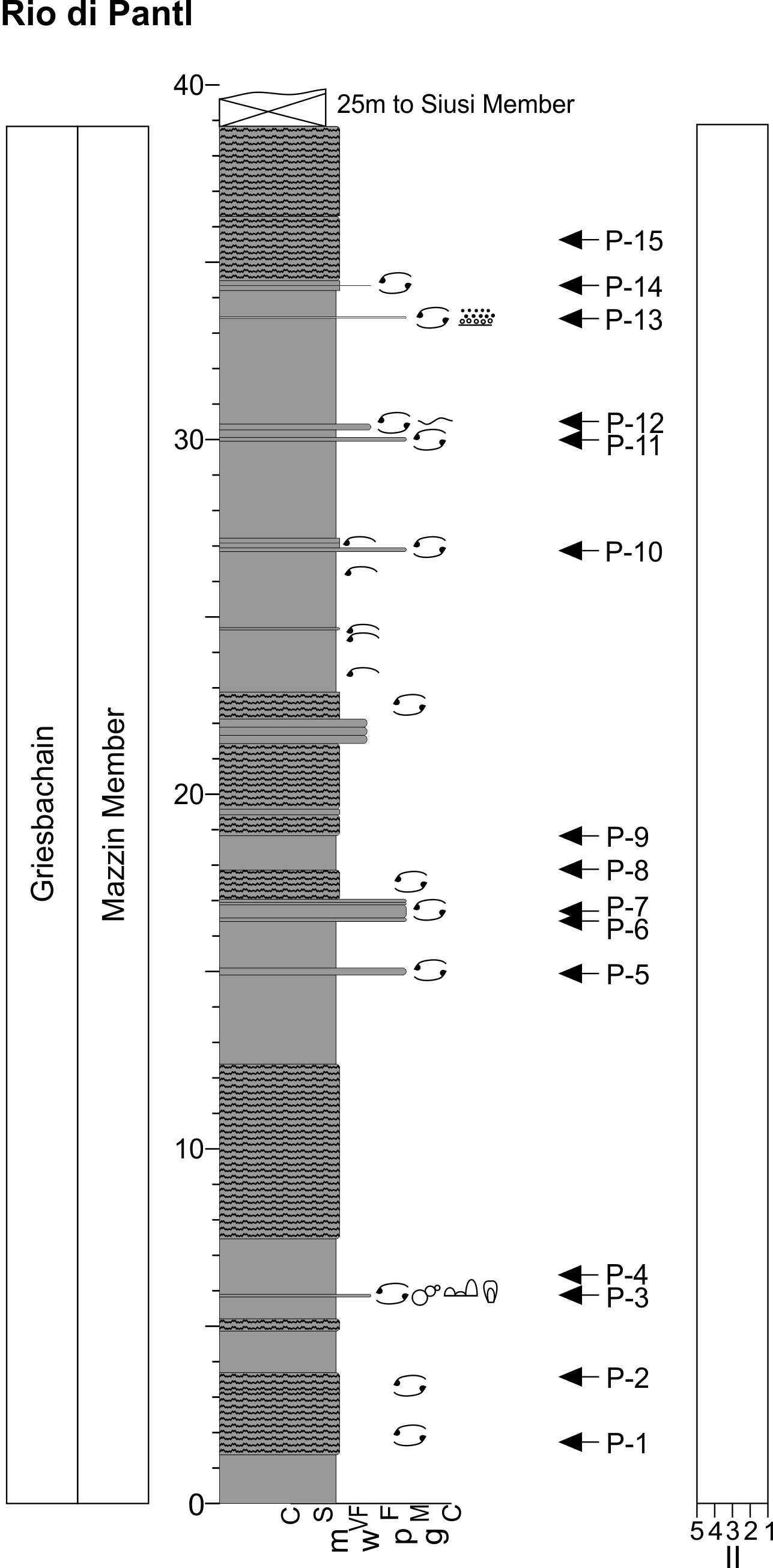

Supplement: S3 Fig — Lithostratigraphy after [24]. For key see S1 Fig. (TIF) [file pone.0172321.s003.tif]

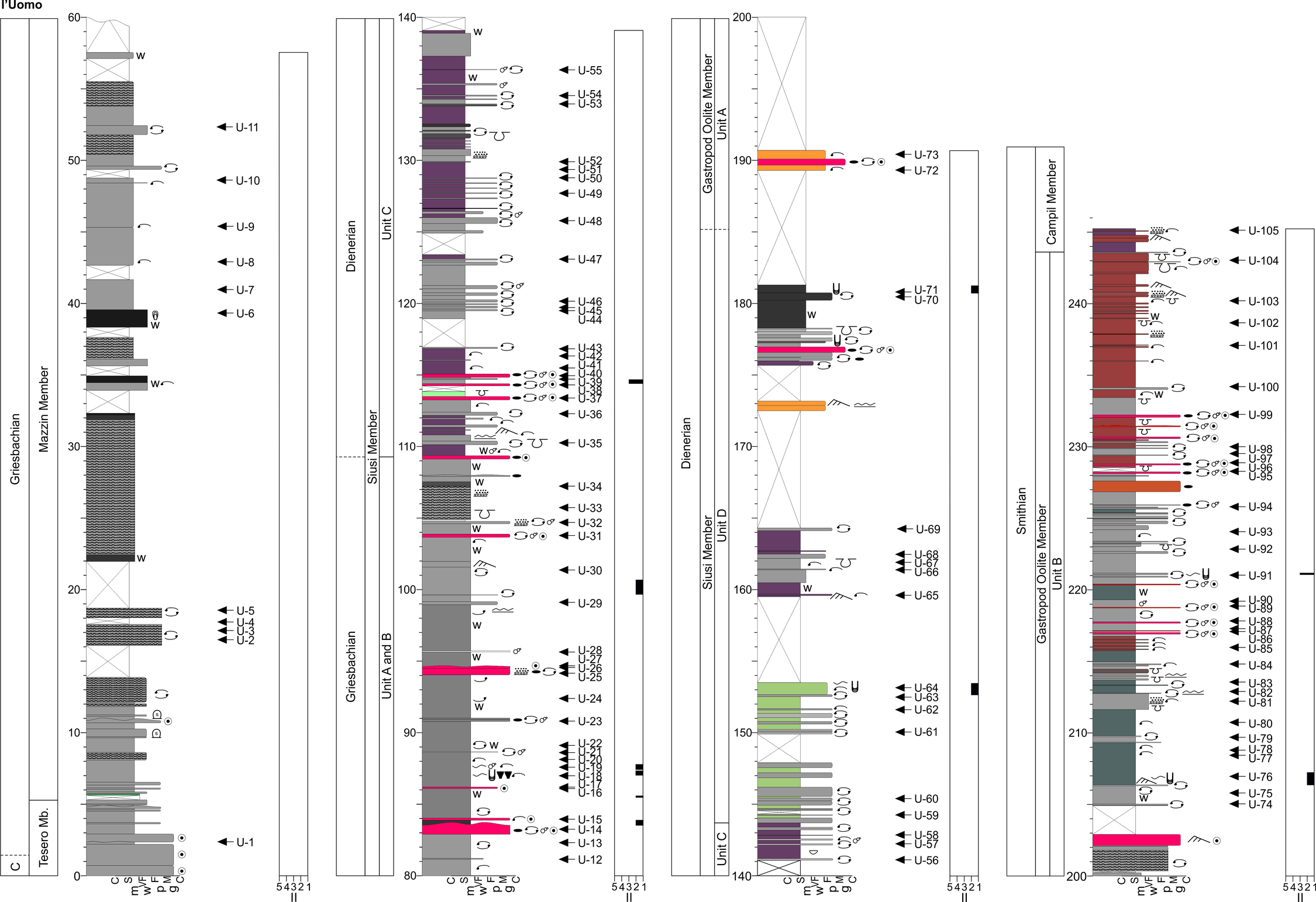

Supplement: S4 Fig — Lithostratigraphy after [24]. For key see S1 Fig. (TIF) [file pone.0172321.s004.tif]

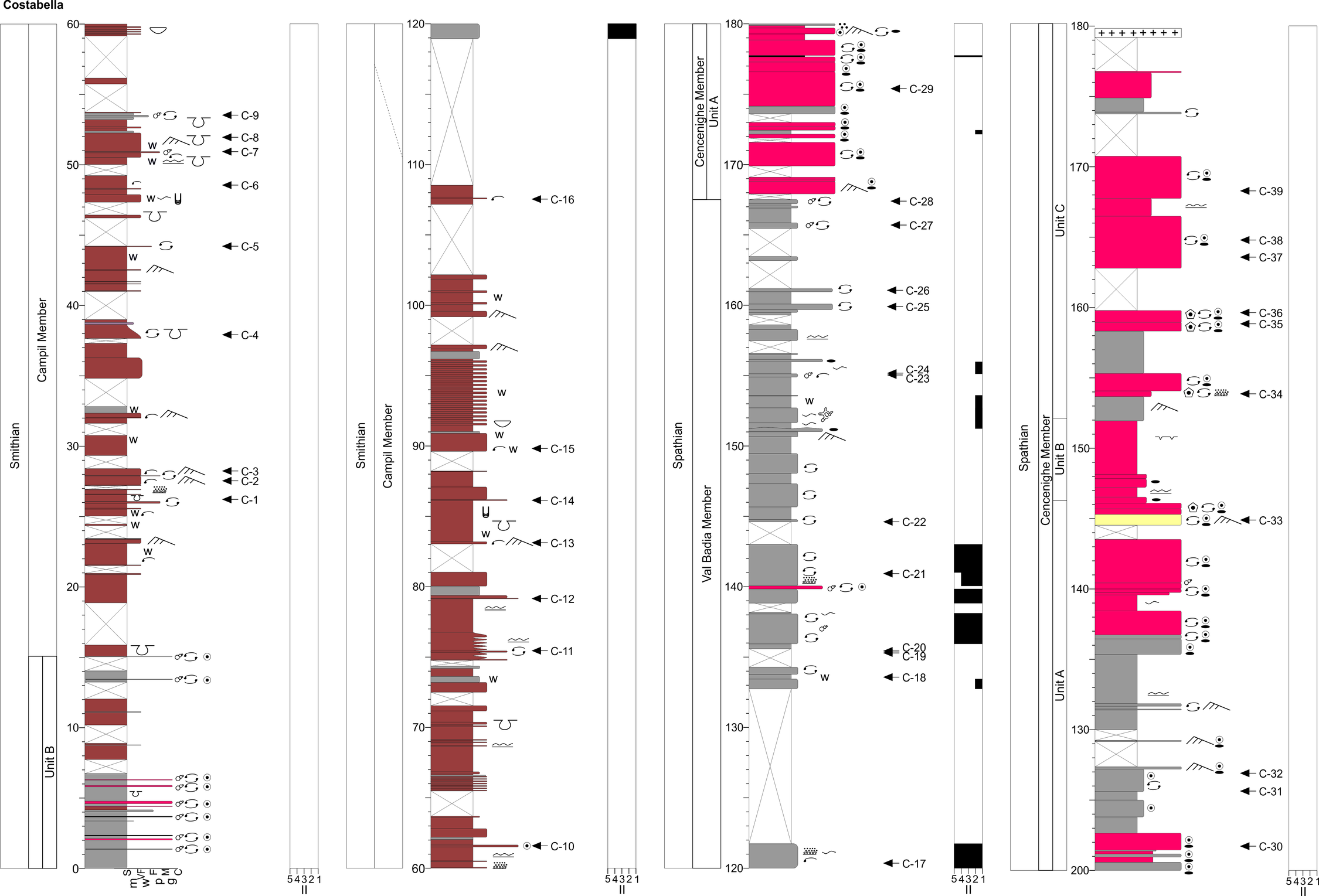

Supplement: S5 Fig — Lithostratigraphy after [24]. For key see S1 Fig. (TIF) [file pone.0172321.s005.tif]

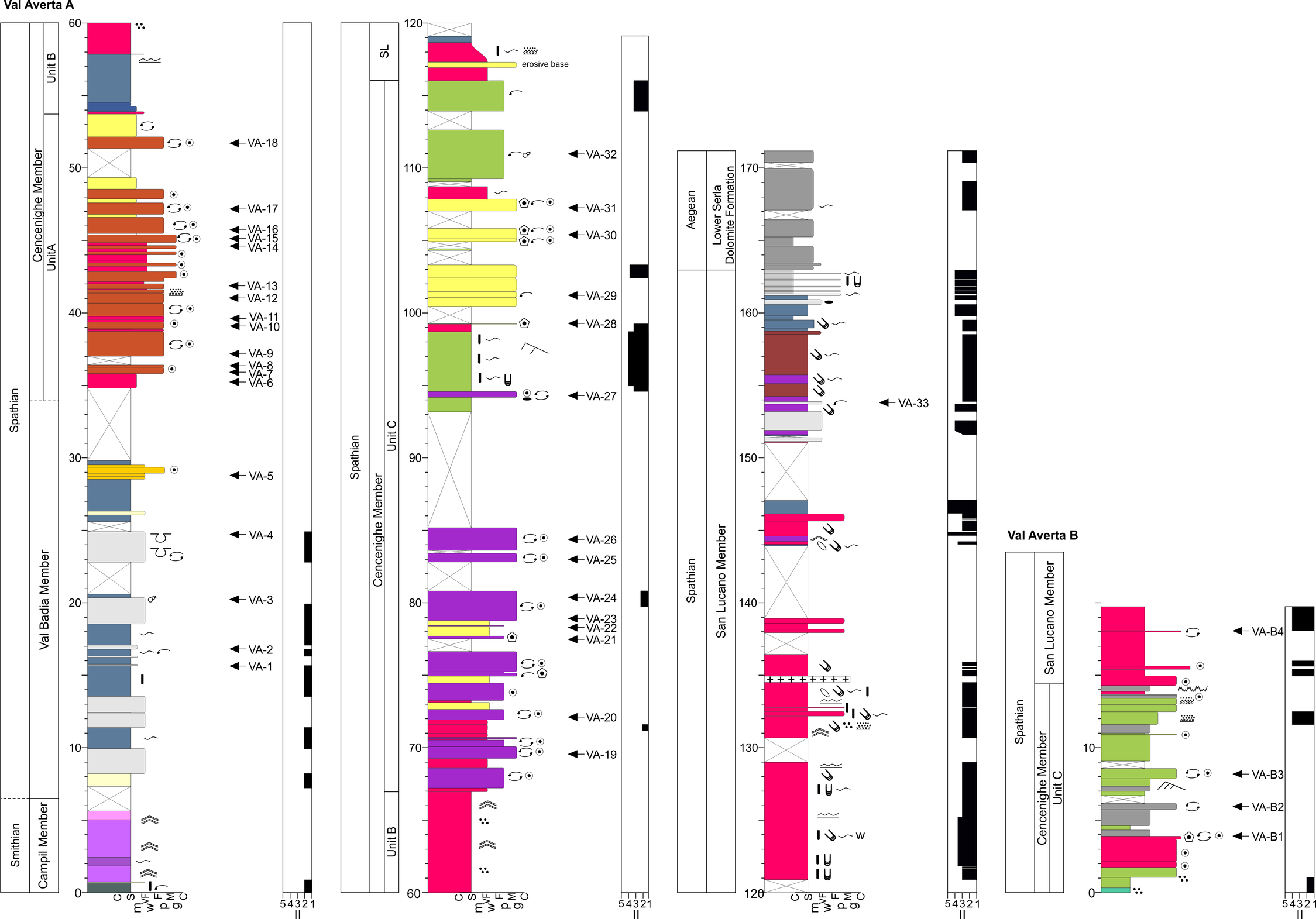

Supplement: S6 Fig — Lithostratigraphy after [24]. For key see S1 Fig. (TIF) [file pone.0172321.s006.tif]
