# Supplementary material for: Subsequent biotic crises delayed marine recovery following the late Permian mass extinction event in northern Italy
Source: PLoS One. 2017 Mar 15;12(3):e0172321. doi: 10.1371/journal.pone.0172321 (PMC5351997; doi:10.1371/journal.pone.0172321)
Supplement: S1 Table — (PDF) [file pone.0172321.s007.pdf]

Table S1: Summary of sedimentary facies of the Werfen Formation, Dolomites.

Facies	Lithology	Sedimentary stuctures and fossils	Depositional Environment
1	Red siltstones	Tepee structures.	Supratidal
2	Red and bluish grey siltstones alternating with varicoloured sandstones.	Siltstones: laminated, bioturbated (ii2) by small stuffed burrows, *Skolithos* and *Planolites*<br>Sandstones: wavy and flaser bedding.	Peritidal
3.1	Red and brown sandstones alternating with grey red siltstones, interbedded by grey packstones.	Siltstones: laminated or bioturbated (ii2-3) by *Diplocraterion*, *Planolites* and *Skolithos*<br>Sandstones: cross-stratification, load structures and ripples.	Shallow subtidal, inner shelf
3.2	Grey wackestones interbedded by grey siltstones.	Siltstones: bioturbated (ii3-5) by *Skolithos*, *Planolites*, *Diplocraterion* and *Rhizocorallium*.<br>Wackestones: randomly orientated fauna and ripples.	Shallow subtidal, inner shelf
3.3	Red siltstones interbedded by fine sandstones.	Siltstones: alternations of laminations with wrinkle marks and bioturbated (ii2-4) by *Rhizocorallium*, *Skolithos*, *Planolites*,	Shallow subtidal, inner shelf
3.4	Green siltstones interbedded with grainstones.	Siltstones: planar-laminated<br>Grainstones: contains ooids, crinoids and convex-up bivalves.	Shallow subtidal, intershoal areas, inner ramp
4.1	Grey oolitic limestones separated by thin green marls.	Packstones and grainstones contain ooids, algae, foraminifera, gastropods, microconchids, bivalves and brachiopods.	Oolitic shoal
4.2	Purple oolitic grainstones.	Thick beds (19-206cm) with hummocky tops. Contains ooids and randomly orientated fauna: bivalves, crinoids ossicles, microconchids and bivalves. Gastropod chambers filled with smaller gastropods or ooids. Bivalves are recrystallised with thick black shells.	Oolitic shoal
4.3	Brown oolitic grainstones.	Thick beds (up to 200cm). Contains ooids and randomly orientated fauna: bivalves, ophiuroids, microconchids and rare crinoids.	Oolitic shoal

5.1	Grey oolitic pack- and grainstones.	5-20cm beds with hummocky tops and erosive bases. Contains: ooids with the nuclei replaced by rhomboidal dolomite, gastropods, shell fragments and convex-up bivalves.	Oolitic shoal storm sheets
5.2	Red-pink oolitic pack- and grainstones	10-63cm beds with hummocky tops and graded in the upper few centimetres. Contains ooids, flat pebbles, randomly orientated fauna including bivalves, microconchids, ostracods and shelly fragments. Gastropod chambers filled with smaller gastropods or ooids.	Oolitic shoal storm sheets
5.3	Brown oolitic pack- and grainstones and fine grained red sandstones.	Contains: ooids, flat pebbles, randomly orientated bivalves.	Oolitic shoal storm sheets
6.1	Grey silty mudstones interbedded by packstones.	Mudstones: Bioturbated (ii2) in the Mazzin Member by *Planolites* Bioturbated (ii2) in the lower Siusi Member by *Thalassinoides*, *Lockeia*, *Diplocraterion*, *Catenichnus* and *Planolites*. Wrinkle marks on bedding planes.<br>Packstones: randomly orientated bivalves, microconchids, ostracods, lingulids, gastropods and ophiuroids.	Mid-shelf with storm sheets
6.2	Grey silty mudstones interbedded by packstones.	Mudstones: Bioturbated (ii3-5) by *Thalassinoides* and *Planolites*.<br>Packstones: randomly orientated bivalves, microconchids, ostracods, lingulids, gastropods and ophiuroids	Mid-shelf with storm sheets
6.3	Brown and grey siltstones alternating with red packstones	Siltstones: laminated, convex-up bivalves, wrinkle marks and load structures.<br>Packstones: rippled tops and gutter casts at their bases. Contains ooids, gastropods and randomly orientated bivalves.	Mid-shelf with storm sheets
6.4	Grey siltstones interbedded with packstones	Siltstones: laminated or weak bioturbation (ii2) by *Skolithos* and *Planolites*. Load structures. Gastropods and convex-up bivalves.<br>Packstones: flat pebbles, ophiuroid ossicles, randomly orientated bivalves.	Mid-shelf with storm sheets
7	Dark grey carbonate mudstones	Stylolaminated or stylonodular bedding, erosive bases, randomly orientated bivalves.	Outer ramp ‘debris flows’
