# Supplementary material for: Subsequent biotic crises delayed marine recovery following the late Permian mass extinction event in northern Italy
Source: PLoS One. 2017 Mar 15;12(3):e0172321. doi: 10.1371/journal.pone.0172321 (PMC5351997; doi:10.1371/journal.pone.0172321)
Supplement: S1 Text — (PDF) [file pone.0172321.s013.pdf]

Supplementary material for Figure 3. Species number on the x-axis are: 1 – Bivalve sp.A, 2 – Bivalve sp.C, 3 – Bivalve sp.B, 4 – *Claraia wangi-griesbachi*, 5 – *Claraia aurita*, 6 – cf. *Unionites danocinus*, 7 – *Claraia clarai* group, 8 – *Neoschizodus laevigatus*, 9 – *Eumorphotis* spp., 10 – *Avichlamys tellinii,* 11 – *Scythentolium* sp., 12 – *Austrotindaria* spp., 13 – *Neoschizodus ovatus*, 14 – *Bakevellia* spp., 15 – *Costatoris costata*, 16 – Gastropod sp.C, 17 – *Warthia vaceki*, 18 – Bellerophotidae with costae, 19 – cf. Worthenia sp., 20 – *Polygyrina* sp., 21 – Gastropod sp.B., 22 – *Werfenella rectocostata*, 23 – Gastropod sp.A, 24 – *Coelostylina werfensis* and *Pseudmurchisonia kokeni*, 25 – *Natiria costata*, 26 – *Allocosmia* sp., 27 – cf. *Plagioglytpa* sp., 28 – Microconchids, 29 – Ostracods, 30 – Ophiuroidea, 31 – *Holocrinus* sp., 32 – Brachiopod sp.A, 33 – *Lingularia* spp.
