# Supplementary material for: Subsequent biotic crises delayed marine recovery following the late Permian mass extinction event in northern Italy
Source: PLoS One. 2017 Mar 15;12(3):e0172321. doi: 10.1371/journal.pone.0172321 (PMC5351997; doi:10.1371/journal.pone.0172321)
Supplement: S2 Text — (PDF) [file pone.0172321.s014.pdf]

Supplementary Material

Polished slab taxonomy

Specimens that could be confidently identified on bedding surfaces of carbonate and sandstone beds were used as reference material, and cut along the transverse and sagittal plane to reveal information on the shell shape, thickness, composition, and ornamentation when viewed in cross-section. These features were then used to discriminate between different taxa observed in the polished slab samples (see below). Shell fragments observed in the polished slabs were identified as such and not included in the analysis. Transverse sections of high-spired gastropods could not be assigned to the different high-spired gastropod morphologies and were identified as such, but excluded from the analysis. Identifications of taxa on the same beds in the field and on the surfaces of the polished slabs themselves were also used to assist with two-dimensional identifications. The identifications of taxa in the Dolomites are comparable to those made in the nearby Lower Triassic succession of the Aggtelek Karst (Foster et al., 2015). Sections of fossils that were only observed in polished slab and not on bedding planes were only identifiable to genus-level at best, e.g. cf. *Microconchus,* based on observations of thin sections and polished slabs in previous Lower Triassic studies (e.g. Nützel and Schulbert, 2005; Foster et al., 2015).

Using the polished-slab technique does include some uncertainty because discriminating between some species is not possible from a two-dimensional view, e.g. *Austrotindaria antiqua* and *Austrotindaria? canalensis.* The specimens identified from the polished slab technique were, therefore, identified to the most precise taxonomic level to which they could be confidently assigned, e.g. *Austrotindaria* was not identified beyond genus-level.

Polished Slab Taxonomy

Gastropods

***Allocosmia* sp.**

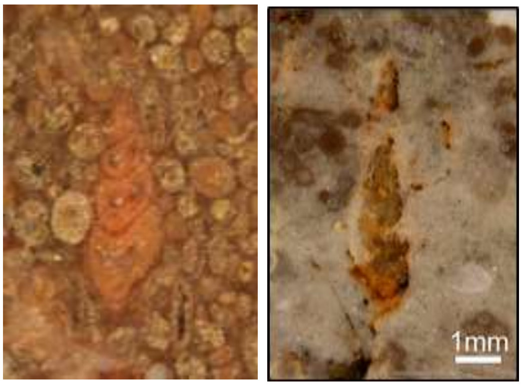

Description

High-spired gastropod, whorls are oval-shaped, with a high-amount of overlap between the whorls, that slowly increase in size, and tightly coiled giving an elongated profile. The shell is thin and smooth. Dextral.

Remarks

Reference material for this morphology was not observed, but these specimens occur in the same beds in the Cencenighe Member at the Val Averta section as those investigated by Posenato (1989). Posenato identified the high-spired gastropod *Allocosmia* which has a similar elongated profile with oval-shaped whorls. The *Allocosmia* specimens differ from Gastropod sp. B with the whorls in *Allocosmia* increasing in size more rapidly, and differ from *Polygyrina* sp. in having a more elongated profile and less well-rounded whorls.

Coelostylina werfensis

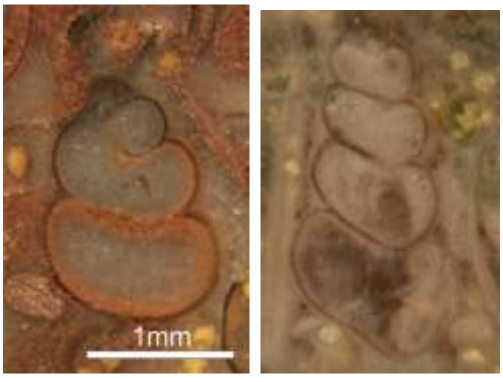

Description

High-spired gastropod, whorls are evenly rounded, smooth, do not overlap, tightly coiled, and rapidly increasing in size. The shell is thin with a layered shell structure with the outer layers thinner than the central layer. Dextral.

Remarks

Coelostylina werfensis has previously been described as occurring in assemblages with '*Polygyrina gracilior*' and *Pseudomurchisonia kokeni* in the Werfen Formation (e.g. Nützel and Schulbert, 2005). Even when present in the same samples, this species can be differentiated from '*Polygyrina gracilior*' in having a less elongated profile (i.e. less high-spired) and by having more rounded whorls, and from

Pseudomurchisonia kokeni in lacking a subsutural ramp. The morphology of this gastropod is comparable to that of *C. werfensis* identified in thin section and reference material of Nützel and Schulbert (2005) and Foster et al. (2015).

Pseudomurchisonia kokeni

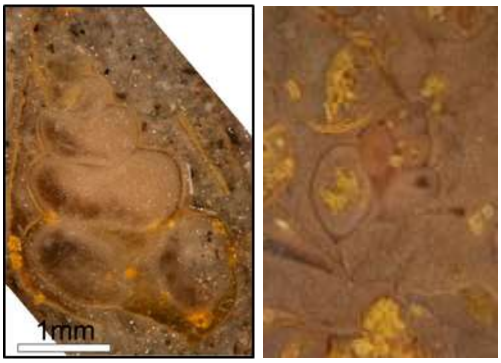

Description

High-spired gastropod, whorls are rounded, tightly coiled, smooth, do not overlap, and rapidly increasing in size. When cut through near the columella, small subsutural ramps are observed at the top the whorls. The shell is thin with a layered shell structure with the outer layers thinner than the central layer. Dextral.

Remarks

Pseudomurchisonia kokeni can be distinguished from *Coelostylina werfensis* by the presence of subsutural ramps (Nützel and Schulbert, 2005). When there is a shallow cut of the whorls, however, they appear smooth and round thus indistinguishable from *C. werfensis*. In this this study, because both species have been reported in the Werfen Formation, and from the same beds, the two species were combined in the identifications to avoid misidentifications. The morphology of this gastropod is also comparable to *Pseudomurchisonia kokeni* identified in thin section and reference material of Nützel and Schulbert (2005).

***Polygyrina* sp.**

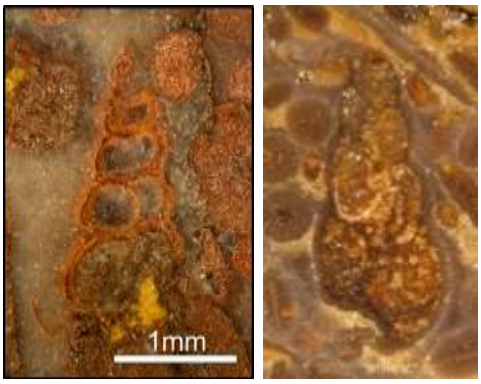

Description

High-spired gastropod, whorls are evenly rounded, some overlap, tightly coiled and slowly increase in size giving an elongated profile. The shell is thin and smooth. Dextral.

Remarks

This species is also identified on the surface of bedding planes. The morphology of *Polygyrina* sp. can be distinguished from *Coelostylina* and *Pseudomurchisonia* as the whorls do not rapidly increase in size, thus giving a more elongated profile. '*Polygyrina gracilior'* has been identified from the Werfen Formation, but has become a dustbin taxon for smooth high-spired gastropods, therefore, these

specimens are only identified to genus-level. This morphology is comparable to that of *'Polygyrina gracilior'* identified in thin section and reference material of Nützel and Schulbert (2005), which supports our identification.

Gastropod sp. A

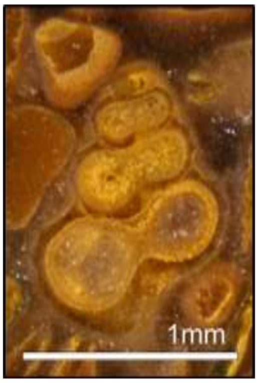

Description

High-spired gastropod, whorls are evenly rounded, do not overlap, tightly coiled, and rapidly increase in size. Thick, three-layered shell structure. The outer shell layer is ornamented with small, evenly spaced acute ribs. Dextral.

Remarks

Ornamented high-spired gastropods have not been reported from the Lower Triassic of Europe and were only observed in the polished slabs in this study. The position of the costae appears to reflect spiral costae and combined with the overall specimen shape are similar to those present in the Early Triassic species *Coelostylina costata* (Batten and Stokes, 1987). This morphology is easily distinguishable from other high-spired gastropods identified in this study by the presence of acute ribs.

Gastropod sp. B

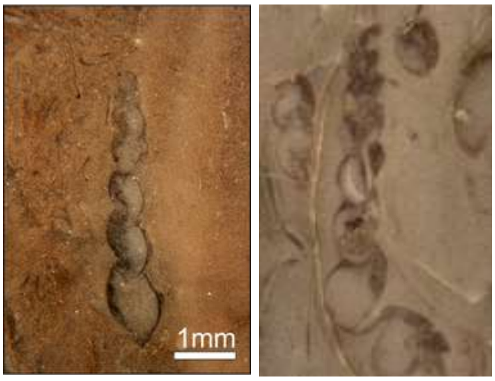

Description

High-spired gastropod, whorls are oval-shaped, high amount of overlap, loosely coiled, and slowly increase in size giving an elongated profile. The shell is thin and smooth. Dextral.

Remarks

This gastropod morphology was not observed in the reference material and has a similar morphology to *Polygyrina* sp. and *Allocosmia* sp. This morphology differs to both these taxa, however, by having oval shaped whorls rather than evenly rounded circular whorls. These specimens also differ to *Polygyrina* and *Allocosmia* in having a more elongated profile (i.e. more high-spired). This morphology also differs to any previous described gastropods from the Werfen Formation. Given the similar morphology with *Polygyrina* sp. this taxon is interpreted to represent a similar mode of life.

cf. *Worthenia* sp.

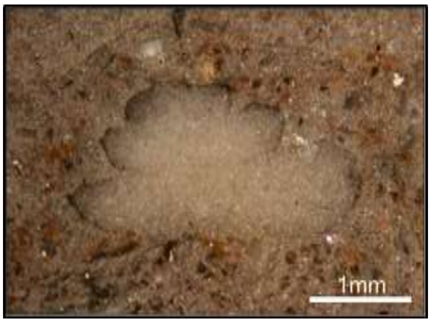

Description

Low-spired gastropod, whorls are rounded with a narrow concave band in the upper third of the whorl with acute ribs on each side. The shell is thin, and the whorls rapidly increase in size. Dextral.

Remarks

Worthenia was not identified in bulk samples or on bedding planes in this study, but has been previously identified from the Werfen Formation (Hofmann et al., 2015). The *Worthenia* sp. specimens figured by Hofmann et al. (2015) have similar ornamentation to that described here, but are high-spired and are, therefore, not considered to be the same species. The narrow concave band in the upper third of the whorl is also similar to *Glabrocingulum*, which is rarely identified in the Triassic (Foster et al., 2016). These specimens may, therefore, represent either *Worthenia* or *Glabrocingulum* and the identification is left in open nomenclature.

Warthia vaceki

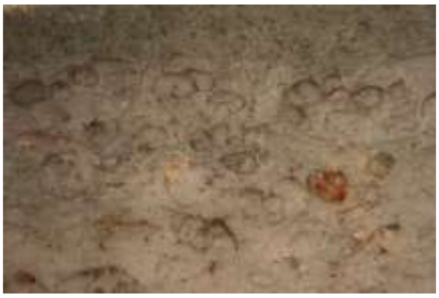

Description

Low-spired, planispiral gastropod; whorls are well-rounded and smooth. Shell is thin, and the whorls rapidly increase in size. When cut along the sagittal plane this gastropod has a bilateral symmetry, and the larger whorl is arched over a smaller whorl that is circular in shape.

Remarks

In the same beds poorly preserved bellerophontids can be observed which have been assigned to *Warthia vaceki* (Kaim and Nützel, 2011). Other *Warthia* species were not recorded in this study and have not been recorded from the Dolomites (e.g. Kaim and Nützel, 2011). The bilateral symmetry associated with bellerophontids is rarely seen in the polished slabs.

Bellerophontidae with costae sp.

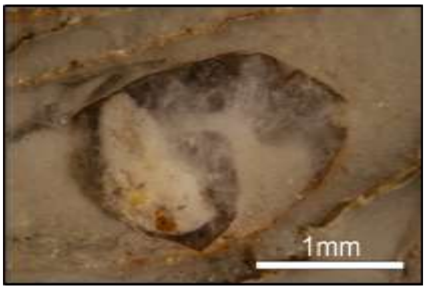

Description

Low-spired, planispiral gastropod; whorls are well-rounded with small acute ribs. The shell is thin, and whorls rapidly increase in size.

Remarks

These specimens are comparable to *W. vaceki* in polished slabs, except they possess small acute ribs which support their separation. Bellerophontid gastropods with costae typically belong to either *Bellerophon, Retispira* or *Dicellonemia*. However, since none of these genera have previously been recorded from the Werfen Formation (previous identification of *Bellerophon* from the Werfen Formation have been reassigned to *Warthia;* Kaim and Nützel, 2011) or nearby sections the identification is not beyond the family-level.

Natiria costata

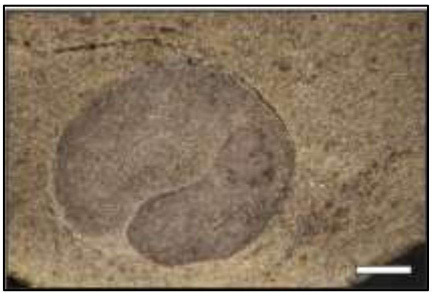

Description

Medium-spired gastropod shell, typically only 1-2 whorls are observed in polished slab, the smaller of the two whorls is more circular whilst the larger whorl is arched around the preceding whorl. Small, evenly spaced fine acute ribs are present on most specimens around the edge of the whorls. Shell is thin, consisting of a single layer. Dextral.

Remarks

A medium-spired gastropod shell in the Dolomites is unique to *Natiria costata* and the ornamentation observed in polished section supports the assignment to this species. In addition, from some of the same beds *N. costata* were observed to be weathering out. This morphology is also comparable to *Natiria costata* identified in the Aggtelek Karst (Foster et al., 2015), which supports our identification.

Bivalves

***Bakevellia* spp.**

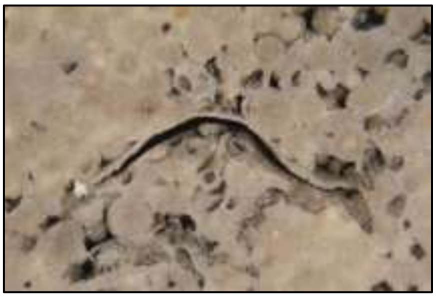

Description

Relatively large bivalve shell; highly convex towards the anterior margin; anterior margin almost becomes flat; posterior margin gently sloping to almost flat. When cut along the sagittal plane the shell is highly convex at the dorsal margin and gently slopes towards the ventral margin. Thick shell with a thin outer layers.

Remarks

These specimens have a characteristic shell form for bivalves with anterior and posterior wings that readily differentiate this genus from others identified in this study. Two genera with this shell form have been recorded from the Werfen Formation: *Bakevellia* and *Pteria* (e.g. Neri and Posenato, 1985; Hofmann et al., 2015). *Pteria*, however, was not observed in this study and these specimens agree well with our reference material of *Bakevellia* cf. *albertii* and are, therefore, assigned to the genus *Bakevellia*. The shell morphology is also indistinguishable from the reference material of *Bakevellia* cf. *incurvata* from the Szin Marl Formation, Hungary (Foster et al., 2015). *Bakevellia* is, however, a diverse genus in the Werfen Formation (e.g. Neri and Posenato, 1985) and so species-level identifications were not made.

***Claraia aurita* group**

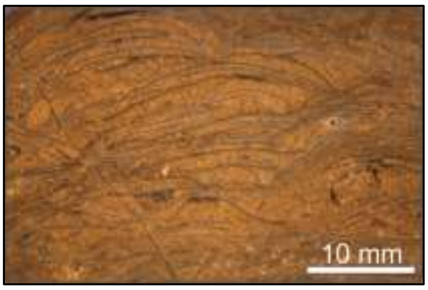

Description

Thin bivalve shell, slightly to moderately convex with no ornamentation.

Remarks

This morphology comprises two species, *Claraia wangi-griesbachi* and *Claraia aurita*, that are differentiated based on their stratigraphic occurrence. In polished slab *Claraia wangi-griesbachi* is indistinguishable from *C. aurita*. The ranges of these two species have, however, not been recorded to overlap in central Europe (Posenato, 2008). Specimens with this morphology that occur in the Mazzin Member are, therefore, assigned to *C. wangi-griesbachi*, whereas those from the Siusi Member are

assigned to *C. aurita*. *Claraia aurita* differs from *C. wangi-griesbachi* in being significantly larger. Hofmann et al. (2015) highlighted that size is not a standard taxonomic character, and so for the palaeoecological analyses these two species are combined as the *C. aurita* group. The bulk samples from this study show for the first time that the ranges of *C. wangi-griesbachi* and *C. clarai* overlap in the Mazzin Member. The morphology of *C. clarai* specimens in polished slabs differ from *C.aurita* group in that the former has visible folds in the shell, whereas in the latter they are absent.

***Claraia clarai* group**

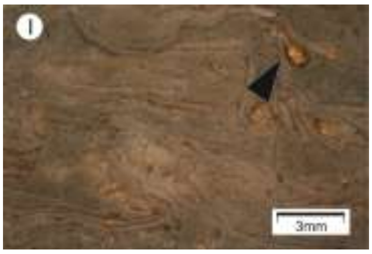

Description

Thin bivalve shell, slightly convex, with regularly spaced folds along the sagittal plane. Along the axial plane small rounded ribs can be observed as well as one or two small folds.

Remarks

The folds observed in *Claraia clarai* and *C. stachei* specimens from both the reference material and the polished slabs make this morphology distinguishable from the other taxa. These specimens clearly differ from *Scythentolium* in having visible folds and possessing a thinner shell. In the polished slabs the reference material for *Claraia clarai* is indistinguishable from *C. stachei*, as the characters that are typically used to distinguish these taxa, such as number of radial ribs, cannot be assessed in a two-dimensional view. Specimens recorded in the polished slabs are, therefore, combined as the *Claraia clarai* group.

***Eumorphotis* spp.**

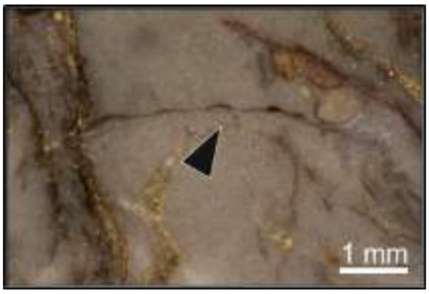

Description

Thin bivalve shell, slightly to moderately convex. Has a three-layered shell structure with each layer having a similar thickness. Ornamentation consists of evenly spaced rounded ribs, unless the valve is cut along the sagittal planes in which case the ornamentation may appear unevenly spaced or occasionally the valve appear smooth.

Remarks

Eumorphotis is a diverse genus in the Werfen Formation (e.g. Broglio Loriga and Mirabella, 1986) and distinguishing between the different species identified in the Dolomites using polished slabs is not possible. *Eumorphotis* differs from *Bakevellia* in lacking a steeply sloping anterior margin and from *Scythentolium* in having a thinner shell and rounded ribs. The genus *Leptochondria* has been reported from the Werfen Formation (Hofmann et al., 2015) but because it was not recorded on the bedding planes or in the mechanically disaggregated samples, and even though in the polished slabs these genera

are likely to have a comparable two-dimensional morphology, these specimens are assigned to *Eumorphotis*. Previously, *Eumorphotis* has been variably placed within the Heteropectinidae (Hofmann et al., 2014), Etheripectinidae (Posenato et al., 2005; Hautmann et al., 2011) or Aviculopectinidae (Carter, 1990; Ros-Franch et al., 2014). The shell structure differs from the calcitic shell structure of the Heteropectinidae figured by Newell and Boyd (1995) and does not resemble any of the variable shell structures of the genera in the Aviculopectinidae (Carter, 1990). The shell structure of the Etheripectinidae has not been described and no comparison is yet possible.

***Neoschizodus* spp.**

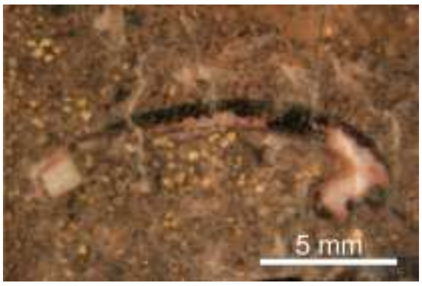

Description

Medium-sized bivalve shell, moderately convex, smooth, with a thick shell composed of three layers. The middle layer is the thickest and the outer and inner layers are very thin.

Remarks

Distinguishing between the reference material for *Neoschizodus laevigatus* and *N. ovatus* was not possible in polished section. In addition, previous studies have also identified other *Neoschizodus* species in the Dolomites (e.g. *N. orbicularis*; Hofmann et al., 2015). A more specific identification was, therefore, not made. The thickness and convexity of the *Neoschizodus* specimens make them clearly distinguishable from *Austrotindaria* and *Unionites* in this study.

***Scythentolium* sp.**

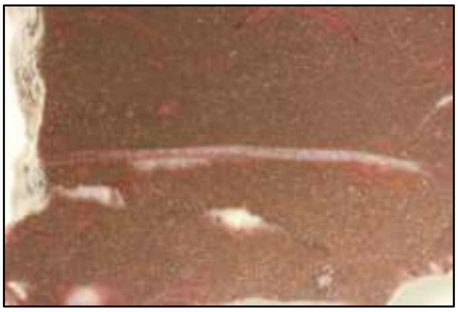

Description

Relatively large bivalve shell, smooth, slightly convex with a thick shell divided into three layers with a thick middle layer and thinner inner and outer layers.

Remarks

These specimens and reference samples lack the ornamentation of *S. tirolicum*. The smooth morphology and slightly inequilateral shell is more similar to *Scythentolium* sp. A figured by Hofmann et al. (2015). The shell structure of entoliids is poorly known (Carter, 1990), and Carter (1990) report that the right and left valves have different microstructures. No apparent differences between valves were observed in this study, which may indicate that only left valves were recognised. The shell structure of *Scythentolium* has not previously been described but the thick middle layer and thin outer layers present in these specimens correspond to Group 1 of Mesozoic Entoliids (after Waller, 2006). This morphology

is also comparable to *Scythentolium* sp. described from the polished slabs of the Aggtelek Karst (Foster et al., 2015).

cf. *Unionites donacinus*

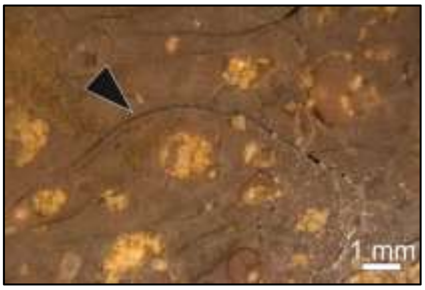

Description

A large bivalve shell, highly convex, smooth, with a variable size. Shell structure consists of a single layer.

Remarks

This morphology is similar to *Austrotindaria*, but differs in being more convex and typically much larger. In addition, the specimens are in good agreement with the reference material for cf. *Unionites donacinus*.

***Austrotindaria* spp.**

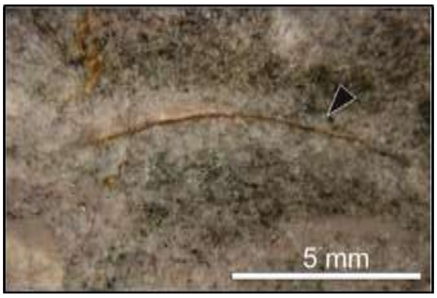

Description

A small bivalve shell, slightly convex, smooth, with a variable size. Shell structure consists of three layers, with the structure of middle layer being more dense than the outer layers.

Remarks

Austrotindaria antiqua and *Austrotindaria? canalensis* were both recorded throughout the Werfen Formation in this study. The shells of these species are both smooth, slightly convex, and there are no features that can be used to distinguish between the two species in cross-section. A more specific identification, therefore, was not possible in this study. The specimens of *Austrotindaria* in this study differ from *Neoschizodus* in having a thinner shell and are less convex. Another species with a similar morphology identified from the Werfen Formation is *Unionites fassaensis*, but it was not recorded in this study and specimens with the morphology described by Wissman (1841) are rarely recorded in the Werfen Formation.

Bivalve sp. A

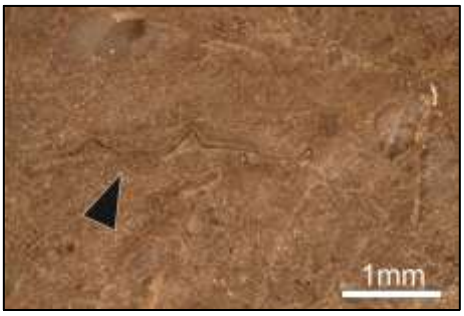

Description

Thin, slightly convex bivalve shell with irregularly spaced, large acute ribs. The ribs are loosely packed along the shell.

Remarks

A bivalve with these features, in particular the large acute ribs, was not observed in the reference material. In addition, bivalves with large acute ribs are currently unreported from the Werfen Formation and other nearby Lower Triassic sections. *Costatoria* has thick acute ribs when observed in polished slab (Foster et al., 2015), however the shell in Bivalve sp. A is much thinner than in *Costatoria*.

Bivalve sp. B

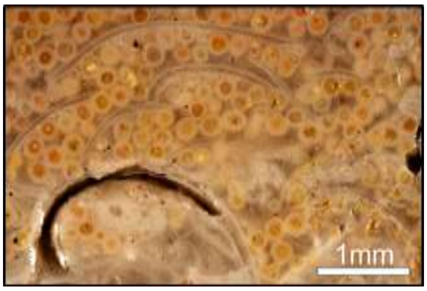

Description

Thin, moderately convex, smooth bivalve shell. The bivalve shell has three layers with the middle layer being the thickest.

Remarks

This bivalve morphology is similar to *Neoschizodus* except that the relative thicknesses of the outer layers of the shell are thicker. This morphology was only observed in the lower part of the Werfen Formation and no comparable specimens were observed on slabs.

Bivalve sp. C

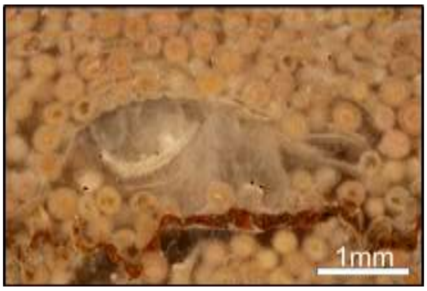

Description

Thin, moderately convex bivalve shell, with small densely packed acute ribs.

Remarks

This bivalve morphology is similar to *Eumorphotis* but it is more convex. The presence of acute ribs, however, means that the morphology can be distinguished from *Eumorphotis*. Another genus with densely packed acute ribs is *Costatoria*. Bivalve sp. C, however, only occurs in the Tesero Member and *Costatoria* is not recorded until the Campil Member in the Werfen Formation (Broglio Loriga and Posenato, 1986). The ribs of *Costatoria* species are also larger than those of Bivalve sp. C, and *Costatoria* species with densely packed acute ribs, e.g. *C. goldfussi* or *C. harpa*, are not recorded until the Middle Triassic. In addition, the shell is thinner than in *Costatoria*.

Other Invertebrates

***Holocrinus* sp.**

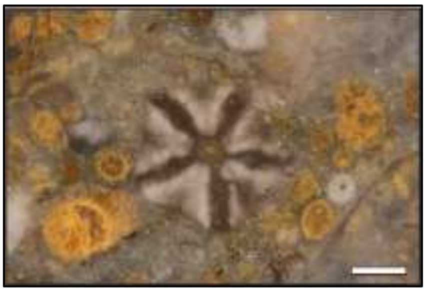

Description

Only preserved as isolated ossicles. If an ossicle is cut transverse to the polished surface it has a pentagonal shape and in some specimens slightly rounded edges. The ossicles have a small circular central lumen, and five petal-like areola areas between the tips of the pentagon and the centre, which are filled with the surrounding matrix. Occasionally, the grooves can be observed as small V shapes between the areola areas. When cut along the sagittal plane, the ossicles appear as rectangles with rounded edges, and if cut along the centre of the ossicle the central lumen is visible.

Remarks

Their pentagonal shape and the presence of *Holocrinus* ossicles from the same beds in the field suggest that these ossicles belong to *Holocrinus*. Other crinoid genera from the Lower Triassic in the western Palaeotethys, including the Dolomites (Hagdorn and Baumiller, 1996) and Aggtelek Karst (Foster et al., 2015), have not yet been recorded. Species-level identifications of *Holocrinus* have not been made for specimens from central Europe although previous authors (Hagdorn and Baumiller, 1996; Hagdorn, 2011) have considered them to represent a separate species than those recorded in Japan (Kashiyama and Oji, 2004) and the western US (Schubert et al., 1992). Other Lower and Middle Triassic crinoid genera, e.g. *Baudicrinus* (Oji and Twitchett, 2015) and *Dadocrinus* (Hagdorn et al., 1997), differ to these specimens as they have a circular, rather than pentagonal, columnal ossicles.

Ophiuroidea

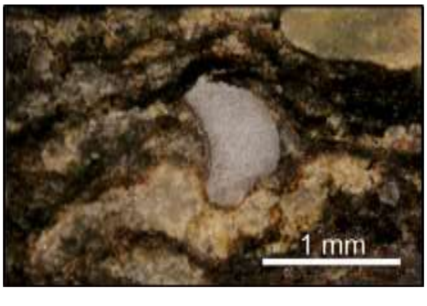

Description

Only preserved as isolated ossicles. Crescent to arched shape, generally with a bilateral symmetry depending on where the polished slab has cut the vertebrae.

Remarks

The only ophiuroid species to be identified from the Lower Triassic of the western Palaeotethys is *Praeaplocoma hessi* (Mostler and Rossner, 1984; Broglio Loriga and Cavicchi, 1972; Hofmann et al., 2015). This species, however, is unknown from non-Spathian strata and without identification of body fossils assigning these ophiuroid ossicles to a species is not possible. Disarticulated ophiuroid ossicles have also previously been reported throughout the Werfen Formation and recorded to dominate some beds (Twitchett et al., 2005).

cf. *Plagioglypta* sp.

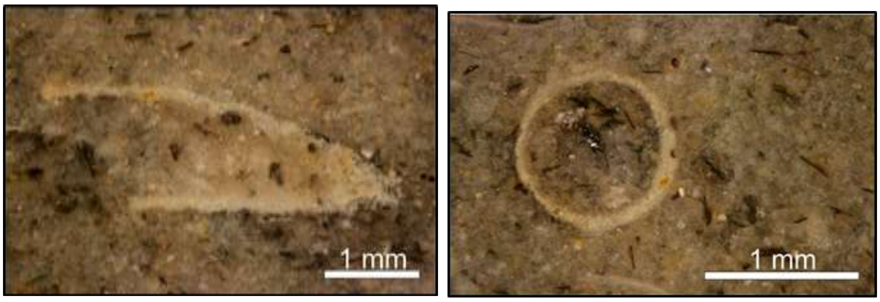

Description

Circular, smooth shell. Shell is composed of one layer. When cut along the sagittal plane it appears as a small cone with small rounded folds.

Remarks

These specimens differ to Dentaliidae sp. identified in the Aggtelek Karst by Foster et al. (2015) in lacking acute ribs which are visible in transverse section, and having small rounded folds when viewed transversely. The reference material appears to have the same morphology as *Plagioglypta* identified from the western US (Nützel and Schulbert, 2005). The occurrence of *Plagioglypta* here represents their first record in the Werfen Formation. These specimens are less slender than the other Lower Triassic scaphopod genus *Laevidentalium*.

Ostracoda

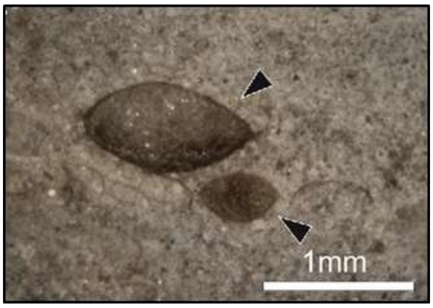

Description

When cut transversely, the shell is kidney-bean shaped, thin and smooth. Along the sagittal plane the shell has an arched shape with a smooth thin shell.

Remarks

A diverse suite of ostracod species have been described from the Bellerophon and Werfen formations (e.g. Crasquin et al., 2008). Observation of their three-dimensional shape, muscle scars and ornamentation are required to distinguish between different ostracod species, which are not visible in

thin section. Identifying the different ostracods to even family level in the polished slabs, therefore, is not possible.

***Microconchus* sp.**

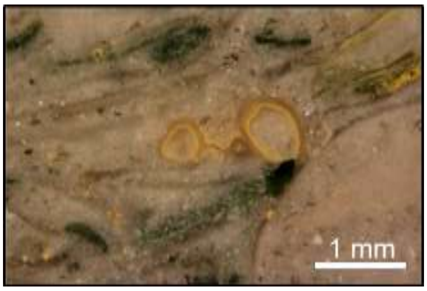

Description

The shell is made up of three to five connected circular tubes with the inner whorl the smallest and successive whorls getting larger, with one flat side by its entire length. Shell wall is distinct and has three layers, with the outer layers being much thinner. Occasionally, when the polished slab cuts through the outer whorl only, these shells appear to have a shallow U-shape. Most specimens do not appear to have been encrusting on anything. Some of the specimens, however, were attached to bivalve shells.

Remarks

This morphology is characteristic of a planispiral microconchid shell. Previous specimens with this morphology from the Dolomites have been identified as the extant polychaete genus *Spirorbis valvata* (e.g. Broglio Loriga and Neri, 1989). These Lower Triassic forms, however, have been shown to have a different microstructure that is more comparable with microconchids (Zatoń et al., 2013). Different genera of microconchids have been distinguished from Lower Triassic thin sections worldwide (e.g. Yang et al., 2015). The forms recognised in this study resemble the tightly coiled Early Triassic species *Microconchus utahensis* and Middle Triassic species *M. valvatus*, a distinction between the two species, however, is not possible in thin section as the ornamentation was not observed. Other Early Triassic species, such as, *Helicoconchus elongates* and *Microconchus aberrans*, differ in being helically coiled to erect (Yang et al., 2015), which was not recorded in these specimens. Microconchids are also easily distinguishable from planispiral gastropods in polished slabs and thin section, with the shell wall being thicker and calcitic, whereas in the planispiral gastropods the shell wall is much thinner and aragonitic. The microconchids also have a flat side that would have been encrusted to a hard substrate (e.g. bivalve shells), whereas encrusting gastropods with a flat side have not been observed from the Werfen Formation.

***Lingularia* spp.**

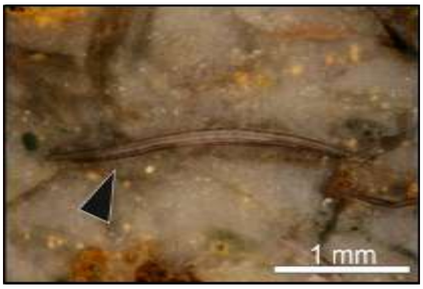

Description

Shell is small, straight, feebly convex, and smooth. Shell has five organophosphatic layers.

Remarks

Multiple *Lingularia* species have been recorded in the Dolomites (e.g. Posenato et al., 2014), and, therefore, a more specific identification could not be made. Other phosphatic inarticulate brachiopods with a similar morphology in polished slab, e.g. *Orbiculoidea*, were not recorded in this study and have not been recorded from the Dolomites in previous studies. The multiple layers of the shell correspond to the thin alternations of mineralised and organic layers of lingulid shells (Iwata, 1981).

References

1. Batten RL. Stokes WL. Early Triassic gastropods from the Sinbad member of the Moenkopi Formation, San Rafael Swell, Utah. American Museum Novitates 1986;2684:1-33.
2. Broglio Loriga C. Cavicchi, A. Praeaplocoma hessi n. gen., n. sp., un Ofiura del Werfeniano (Trias Inferiore) del Gruppo della Costabella, Dolomiti. Memorie Geopaleontologiche dell' Universita di Ferrara 1972;2:185-197.
3. Broglio Loriga C. Mirabella S. Il genere Eumorphotis Bittner 1901 nella biostratigrafia dello Scitico, Formazione di Werfen (Dolomiti). Memorie di Scienze Geologiche 1986;38:245-81.
4. Broglio Loriga C. Neri N. Spirorbis valvata community from Werfen Formation: an example of the Scythian oligotypy (lower Scythian, Southern Alps, Italy). In: Atti 3 Simposio di Ecologia e Paleoecologia delle Comunità bentoniche. Università di Catania, Catania 1989:123-140.
5. Broglio Loriga C. Posenato R. Costatoria (Costatoria?) subrotunda (Bittner, 1901) a Smithian (Lower Triassic) marker from Tethys. Rivista Italiana di Paleontologia e Stratigrafia 1986;92:89-200.
6. Carter JG. Evolutionary significance of shell microstructure in the Palaeotaxodonta, Pteriomorphia and Isofilibranchia (Bivalvia: Mollusca). Skeletal biomineralization: patterns, processes and evolutionary trends 1990;1:135-296.
7. Crasquin SY. Perri MC. Nicora A. De Wever PA. Ostracods across the Permian-Triassic boundary in Western Tethys: the Bulla parastratotype (Southern Alps, Italy). Rivista Italiana di Paleontologia e Stratigrafia 2008;114:233-262.
8. Foster WJ. Danise S. Sedlacek A. Price GD. Hips K. Twitchett RJ. Environmental controls on the post-Permian recovery of benthic, tropical marine ecosystems in western Palaeotethys (Aggtelek Karst, Hungary). Palaeogeography, Palaeoclimatology, Palaeoecology 2015;440:374-394.
9. Foster WJ. Danise S. Twitchett RJ. A silicified Early Triassic marine assemblage from Svalbard. Journal of Systematic Palaeontology 2016; doi/full/10.1080/14772019.2016.1245680.
10. Hagdorn H. Triassic: the crucial period of post-Palaeozoic crinoid diversification. Swiss Journal of Palaeontology 2011;130:91-112.
11. Hagdorn H. Baumiller TK. Distribution, morphology and taphonomy of Holocrinus, the earliest post-Paleozoic crinoid. In: Echinoderms 1998:163-168.
12. Hagdorn H. Torok A. Konrad G. Crinoids from the Muschelkalk of Mecsek Mountains and their stratigraphical significance. Acta Geologica Hungarica 1997;40:391-410.
13. Hautmann M. Bucher H. Brühwiler T. Goudemand N. Kaim A. Nützel A. An unusually diverse mollusc fauna from the earliest Triassic of South China and its implications for benthic recovery after the end-Permian biotic crisis. Geobios 2011;44:71-85.

14. Hofmann R. Hautmann M. Brayard A. Nützel A. Bylund KG. Jenks JF. Vennin E. Olivier N. Bucher H. Recovery of benthic marine communities from the end- Permian mass extinction at the low latitudes of eastern Panthalassa. Palaeontology 2014;57:547-89.
15. Hofmann R. Hautmann M. Bucher H. Recovery dynamics of benthic marine communities from the Lower Triassic Werfen Formation, northern Italy. Lethaia. 2015;48:474-96
16. Iwata K. Ultrastructure and Mineralization of the Shell of Lingula unguis Linne,(Inarticualte Brachiopod. Journal of the Faculty of Science, Hokkaido University. Series 4, Geology and mineralogy 1981;20:35-65.
17. Kaim A. Nützel A. Dead bellerophontids walking—The short Mesozoic history of the Bellerophontoidea (Gastropoda). Palaeogeography, Palaeoclimatology, Palaeoecology 2011;308:190-9.
18. Kashiyama Y. Oji T. Low-diversity shallow marine benthic fauna from the Smithian of northeast Japan: paleoecologic and paleobiogeographic implications. Paleontological Research 2004;8:199-218.
19. Mostler H. Roßner PD. Mikrofazies und Palökologie der höheren Werfener Schichten (Untertrias) der Nördlichen Kalkalpen. Facies 1984;10:87-143.
20. Neri C. Posenato R. New biostratigraphical data on uppermost Werfen Formation of western Dolomites (Trento, Italy). Geologisch− Paläontologische Mitteilungen Innsbruck 1985;14:83-107.
21. Newell ND. Boyd DW. Pectinoid bivalves of the Permian-Triassic crisis. Bulletin of the AMNH; no. 227.
22. Nützel A. Schulbert C. Facies of two important Early Triassic gastropod lagerstätten: implications for diversity patterns in the aftermath of the end-Permian mass extinction. Facies 2005;51:480-500.
23. Oji T, Twitchett RJ. The Oldest Post-Palaeozoic Crinoid and Permian-Triassic Origins of the Articulata (Echinodermata). Zoological Science 2015;32:211-215.
24. Posenato R. Un'Associazione olgitipica a Neoschizodus ovatus (Goldfuss) della formazione de Werfen (Triassico Inf-Dolomiti). In: Atti 3 Simposio di Ecologia e Paleoecologia delle Comunità bentoniche. Università di Catania, Catania 1989:141-153.
25. Posenato R. Global correlations of mid Early Triassic events: the Induan/Olenekian boundary in the Dolomites (Italy). Earth-Science Reviews 2008;91:93-105.
26. Posenato R, Pelikán P, Hips K. Bivalves and Brachiopods near the Permian-Triassic boundary from the Bükk Mountains (Bálvány-North Section, northern Hungary). Rivista Italiana di Paleontologia e Stratigrafia 2005;111:215-232.

27. Posenato R. Holmer LE. Prinoth H. Adaptive strategies and environmental significance of lingulid brachiopods across the late Permian extinction. Palaeogeography, Palaeoclimatology, Palaeoecology. 2014;399:373-84.
28. Ros-Franch S. Márquez-Aliaga A. Damborenea SE. Comprehensive database on Induan (Lower Triassic) to Sinemurian (Lower Jurassic) marine bivalve genera and their paleobiogeographic record. Paleontological Contributions 2014;8:1-219.
29. Schubert JK. Bottjer DJ, Simms MJ. Paleobiology of the oldest known articulate crinoid. Lethaia. 1992;25:97-110.
30. Twitchett RJ. Feinberg JM. O'Connor DD. Alvarez W. McCollum LB. Early Triassic ophiuroids: their paleoecology, taphonomy, and distribution. Palaios 2005;20:213-23.
31. Waller TR. Phylogeny of families in the Pectinoidea (Mollusca: Bivalvia): importance of the fossil record. Zoological Journal of the Linnean Society 2006;148:313-42.
32. Wissman HL. Beiträge zur Geognosie und petrefactenkunde des Südöstlichen Tirol's. Beiträge zur Petrefactenkunde 1841;4:1–152.
33. Yang H, Chen ZQ, Wang Y, Ou W, Liao W, Mei X. Palaeoecology of microconchids from microbialites near the Permian–Triassic boundary in South China. Lethaia 2015;48:497-508.
34. Zatoń M, Taylor PD, Vinn O. Early Triassic (Spathian) post-extinction microconchids from western Pangea. Journal of Paleontology 2013;87:159-65.
